# Ergothioneine-Associated Transcription Opposes Conserved Mitochondrial Injury Programs in Proximal Tubules

**DOI:** 10.3390/antiox15101239

**Published:** 2026-09-25

**Authors:** Yirui Chen, Hongxin Gui, Xingting Xue, Mingyuan Liu, Zixin Zhang, Shibei Tang, Kai Ma, Mengyang Wang, Rongrong Yang

**Affiliations:** 1 School of Public Health and Health Sciences, Tianjin University of Traditional Chinese Medicine, No. 10 Poyanghu Road, Jinghai District, Tianjin 301617, China; ciandata@163.com (Y.C.); ghostcixi@163.com (H.G.); mmingyuanliu@163.com (M.L.); zhangzixin0514@163.com (Z.Z.); tangshibei1229@163.com (S.T.); 2 Department of Electronics, Information and Bioengineering (DEIB), Politecnico di Milano, Piazza Leonardo da Vinci 32, 20133 Milan, Italy; 3 Haihe Laboratory of Modern Chinese Medicine, Tianjin University of Traditional Chinese Medicine, No. 10 Poyanghu Road, Jinghai District, Tianjin 301617, China; xuexingting@126.com (X.X.); yichongma@126.com (K.M.); 4 Department of Neurobiology, Care Sciences and Society, Karolinska Institutet, Tomtebodavägen 18A, Floor 10, SE-171 65 Solna, Sweden

**Keywords:** acute kidney injury, ergothioneine, proximal tubule, inflammatory signaling, mitochondrial bioenergetics, mitochondrial translation, pathway enrichment

## Abstract

Acute kidney injury (AKI) is characterized by tubular epithelial stress, inflammation, and metabolic dysfunction, yet the transcriptional programs associated with effective metabolic recovery remain poorly defined. Ergothioneine (EGT) has demonstrated renal and mitochondrial protective effects, but its potential involvement in conserved tubular injury programs is unclear. Here, we integrated human and mouse transcriptomic datasets to identify injury-associated programs that were conserved across proximal tubules and oppositely associated with EGT treatment. Pathway-level analyses revealed coordinated inflammatory and redox responses in human AKI, while the mouse intervention model showed broad reversal of disease-associated transcription following EGT treatment. Cross-species analysis of 9700 common orthologs identified seven pathways that consistently exhibited human injury-associated, mouse disease-associated, and EGT-opposed enrichment after removal of cytosolic ribosomal genes. These pathways converged on a mitochondrial bioenergetic–translation program involving oxidative phosphorylation, respiratory electron transport, complex I biogenesis, and mitochondrial translation, together with mTORC1 and MYC signaling. Within this conserved program, OXA1L, NDUFAB1, and TUFM repeatedly contributed to multiple pathway leading edges, highlighting them as potential candidates for functional investigation. Collectively, our findings identify a conserved mitochondrial transcriptional program associated with EGT in injured proximal tubules and provide a mechanistic framework for testing whether modulation of mitochondrial bioenergetics and translation contributes to the renal protective effects of EGT.

## 1. Introduction

Acute kidney injury (AKI) is a common global syndrome that affects patients across hospital and critical-care settings and remains an important contributor to subsequent chronic kidney disease (CKD) and mortality [1]. Specific treatments that improve tubular recovery remain limited. Recovery depends on epithelial repair and resolution of inflammation; incomplete repair can instead lead to tubular atrophy and fibrosis [2]. Human kidney atlases have identified several injury-associated epithelial states within distinct tissue neighborhoods [3,4,5]. These states provide a cellular basis for investigating why injured proximal tubules (PTs) follow different courses after an acute insult.

Inflammation and metabolic stress are closely linked features of the injured tubular epithelium. Experimental models distinguish PT states with altered lipid and amino-acid metabolism from states expressing inflammatory and profibrotic programs [6,7]. Adaptive regeneration is associated with fatty-acid oxidation and oxidative phosphorylation, whereas fibrotic repair includes epithelial signals that recruit myeloid cells [8,9]. Redox responses intersect with both processes, but physiological signaling, compensatory antioxidant transcription, and oxidative damage have different biological implications [10,11]. Reactive oxygen species can also impair ribosomal elongation and activate stress signaling, linking oxidative injury to protein synthesis [12]. The molecular programs within this response therefore need to be resolved beyond broad pathway labels.

Interpreting natural bioactive compounds requires attention to their metabolic context. Metabolic activation can generate reactive intermediates that contribute to toxicity, including nephrotoxicity, as reviewed for several classes of traditional Chinese medicine constituents [13]. Ergothioneine (EGT) offers an experimental setting in which to examine treatment-associated changes in renal stress responses. In a mouse cystinuria model, EGT improved renal glutathione metabolism and mitochondrial respiratory capacity [14]. Direct activation of mitochondrial 3-mercaptopyruvate sulfurtransferase by EGT has also been demonstrated in muscle and exercise models [15]. More recently, a cisplatin-induced AKI-to-CKD study reported renal and mitochondrial protection and released single-cell transcriptomic data [16].

Complementary molecular measurements can help resolve organ-injury responses to natural compounds. For example, integrated metabolomic and gut-microbiota profiling linked bavachin–icariside II coadministration to metabolic disturbances in a susceptible mouse liver-injury model [17]. In kidney research, cross-species pathway coordination provides a complementary strategy for prioritizing conserved responses [18]. An unresolved question is which disease-associated transcriptional programs change with EGT, and how much of their apparent breadth reflects genes shared across annotations.

Our primary objective was to identify injury-associated transcriptional programs conserved between human and mouse proximal tubules and oppositely associated with EGT treatment. A secondary methodological objective was to determine how recurrent, highly connected genes influence the apparent breadth of pathway enrichment. We combined human AKI and mouse EGT transcriptomes with complete pathway libraries and a common one-to-one ortholog universe. Gene-membership analyses assessed pathway convergence, while published PT-state and spatial datasets supplied cellular and tissue context.

## 2. Materials and Methods

### 2.1. Public Data and Analysis Units

Data were obtained from the NCBI Gene Expression Omnibus (GEO) [19]. Table 1 summarizes the four datasets, their source publications, and the samples entering each analysis. The human source characterized recurrent epithelial responses in severe AKI [20]. The lineage study profiled adaptive and maladaptive repair using lineage tracing and single-nucleus multiomics [21]. The spatial study mapped acute injury-associated epithelial and immune transcription [22]. The EGT dataset accompanies the intervention study introduced above. Each dataset was analyzed within its own experimental design.

We defined two independent primary analyses for this reanalysis. The human AKI analysis (GSE210622) tests injury-associated transcription in human proximal tubules, and the EGT intervention analysis (GSE329941) tests disease-versus-control and EGT-versus-disease contrasts in mouse proximal tubules. GSE209610 (PT states) and GSE171406 (spatial profiles) were analyzed as contextual datasets: they provide cellular-state and tissue-location context for the primary signatures without being merged with either primary contrast or used as independent replication cohorts. All four datasets were therefore analyzed within their own experimental designs.

GSE210622 contains 12 individuals and 14 libraries: eight individuals with AKI, three nephrectomy controls, and one post mortem control sampled at three intervals. The focal contrast retained a marker-defined injury-associated PT subset from seven AKI samples and the three nephrectomy controls after state-level filtering. The inherited cell-type landscapes and pooled display compositions are shown in Figure A1 as annotation context.

### 2.2. Expression Processing and Differential Contrasts

The recorded single-cell workflow used Scanpy (version 1.12.4) [23]. Cells or nuclei required at least 300 detected genes, and genes at least three cells. Mitochondrial-read thresholds were 10% for GSE210622, 15% for GSE209610, and 20% for GSE329941; upper count and gene outliers were screened at four median absolute deviations. Expression representations used normalization to 10,000 counts, log transformation, 3000 variable genes, 40 principal components, and a 20-neighbor graph. The workflow configured Harmony (harmonypy version 2.0.2) for integrated representations and Scrublet (version 0.2.3) for sample-level doublet screening [24,25]. Pseudobulk models used the source count matrices and biological samples as the replication unit.

Sample–cell-type pseudobulks required at least 30 cells or nuclei. Human and EGT contrasts used edgeR (version 4.10.5; Bioconductor version 3.23) filtering, trimmed-mean-of-M-values normalization, robust dispersion estimation, and quasi-likelihood testing [26,27]. The recorded filterByExpr setting was min.count = 1, with at least two retained libraries per group. In the mouse intervention, all three conditions entered one design matrix and one quasi-likelihood fit per cell type; the disease-versus-control and EGT-versus-disease contrasts were extracted from that fit. GSE209610 RNA counts were aggregated by sample and existing RNA-defined state; filtering used min.count = 10 and min.total.count = 20, followed by a GFP-positive versus control model at four weeks. Biological samples, rather than individual cells, define the replication unit for these contrasts [28]. The lineage analysis retains the published state assignments.

For the human focal population, Leiden clusters at resolution 0.6 were annotated by the highest cluster-mean score among fixed cell-type marker lists. Scores were means of normalized log-expression, and condition labels were not annotation inputs. The injury-associated PT list comprised VCAM1, CCL2, CXCL1, KRT20, CDKN1A, and TGFB2. An additional sensitivity analysis removed these six markers from the human ranking and pathway memberships, then repeated complete-library enrichment. This evaluates their direct contribution to enrichment. Per-sample retained-cell counts are reported in Appendix A.1.

### 2.3. Complete-Library Enrichment

Gene sets were frozen from MSigDB 2026.1 using msigdbr; the MSigDB resource and Hallmark collection are described in their corresponding publications [29,30]. Reactome annotations supplied the second pathway library [31]. The original within-study analyses retained frozen human and mouse pathway memberships, generated through Ensembl BioMart and biomaRt [32,33]. For the cross-species comparison, we used the retained one-to-one human–mouse ortholog table and enforced unique human and mouse symbol assignments before merging the three profiles. The retained mapping is documented with the processed results. All frozen memberships and the mapping used for the comparison are included in the reproducibility materials.

All finite, uniquely mapped gene statistics were ranked as

(1) $$r_{g}=sign({log}_{2}{FC}_{g})\sqrt{F_{g}},$$

where *F_g_* is the edgeR quasi-likelihood statistic. Gene-level significance was not used as a ranking filter. Sets containing 10–500 ranked genes were tested by fgseaMultilevel, with eps = 0 and one worker [34]. Competitive enrichment used limma::cameraPR, with use.ranks = TRUE and an assumed inter-gene correlation of 0.01 [35]. Benjamini–Hochberg (BH) correction was applied separately within each complete library, contrast, analysis mode, and method [36]. Concordant support required adjusted *p <* 0.05 in both methods and the same enrichment direction. This criterion measures agreement between tests of the same data. Normalized enrichment scores (NES) describe ranked enrichment, rather than a biological effect size.

The human ranking contained 12,839 genes and the two EGT rankings 23,923 genes each. A frozen cytosolic-ribosomal list, including canonical RPL/RPS genes and UBA52/FAU but excluding RPS6 kinases, was removed from both rankings and pathway memberships. This removed 80 ranked human genes and 56 ranked mouse genes. Enrichment and full-library correction were repeated after removal. Sets with fewer than ten remaining genes were classified as non-testable. A previously selected 20-pathway panel was used only for the secondary PT-state display; no panel-specific discovery fraction is a primary endpoint here. A focused redox-related gene panel was additionally summarized descriptively in Supplementary Table S2 and did not define the primary pathway analysis.

### 2.4. Pathway Overlap and Sensitivity Controls

We quantified overlap across all positively enriched human pathways and all EGT disease-up/treatment-down pathways satisfying the primary concordance criterion. Pairwise Jaccard similarity was calculated for measured pathway genes and for fgsea leading-edge genes. Complete-linkage clustering of 1 − Jaccard, cut at 0.75, grouped pathways sharing at least 0.25 similarity within each cluster. This descriptive grouping is threshold-dependent. Unique genes, repeated genes, total pathway–gene memberships, and ribosomal membership fractions were tabulated separately. A membership fraction counts a gene once for every pathway in which it occurs and therefore differs from the fraction of unique genes.

CAMERA was implemented with an assumed inter-gene correlation of 0.01. A separate sensitivity pooled Hallmark and Reactome probabilities into one BH family within each contrast, mode, and method. Membership clustering was evaluated at Jaccard thresholds 0.15, 0.20, 0.25, 0.30, and 0.35.

Two removal controls used 200 draws each: matching gene number alone, or matching number and signed ranking strength. Human matching used sign and decile of absolute-statistic rank percentile. Mouse matching used joint signs and quintiles in both EGT contrasts, removing the same genes from both rankings. Sampling was without replacement within a draw, with seed 202609053. Each draw repeated CAMERA at correlation 0.01 and BH correction across the eligible full libraries. These CAMERA-only endpoints complement the dual-method pathway results. The removal endpoint was retention of baseline-supported pathway labels; newly supported labels were recorded separately.

An exploratory control additionally matched annotation degree, defined as the number of baseline-testable Hallmark and Reactome pathways containing each gene. Final calipers required identical statistic signs, absolute-rank percentile differences ≤0.05 in every relevant contrast, and ratios of degree plus one within [1/1.15, 1.15]. Maximum bipartite matching identified fixed common-support subsets of 79 human and 25 mouse ribosomal genes. Unmatched targets remained unchanged in the primary comparison. We generated 1000 randomized feasible assignments per group, retaining only assignments with absolute standardized mean differences ≤0.10 for degree, log-transformed degree plus one, and rank-percentile features (seed 202609057). Calipers were refined using feature-balance diagnostics during this revision; this control was exploratory. A conditional analysis removed unmatched targets in every actual and reference ranking. Appendix A.5 details the restricted reference space and the different questions addressed by these two scopes. The matching variables were limited to the available ranking and pathway-membership features.

The lower-tail reference fraction was calculated as

(2) $$p_{ref}=\frac{1+\sum_{b=1}^{B}1(K_{b}\leq K_{rib})}{B+1},$$

where *K_b_* is the retained baseline-pathway count in reference draw *b*, *K*_rib_ is the count after the corresponding ribosomal removal, and *B* is the number of draws. This quantity summarizes membership sensitivity, not a biological treatment probability. Empirical 2.5th– 97.5th percentiles describe the reference distribution. The vectorized rank-based CAMERA implementation was checked against limma::cameraPR with absolute tolerance 10^−12^.

### 2.5. EGT Contrasts and Existing PT States

The EGT analysis used disease versus control and EGT-treated disease versus un-treated disease contrasts. Opposite-direction enrichment required positive disease NES, negative EGT NES, and concordant support in both contrasts. For every tested PT gene, we also calculated the fitted EGT-versus-control effect estimate

(3) $${\hat{\Delta }}_{g,EGT-control}={\hat{\Delta }}_{g,disease-control}+{\hat{\Delta }}_{g,EGT-disease},$$

where each $\hat{\Delta }$ is a log2 fold change from the common three-group fit. Their sum is the estimated EGT-versus-control log-fold difference. All 23,923 gene estimates are displayed, with zero marking the estimated control reference; this gene-level effect view complements the pathway analysis.

GSE209610 contributed eight existing PT states: C0, C1, C2, C3, C12, C13, C14, and C15. For this secondary analysis, BH correction covered all 20 × 8 = 160 pathway–state tests separately for each enrichment method. The complete heatmap displays within-state enrichment; differences in significance between states do not constitute a formal state-by-condition interaction test.

### 2.6. Cross-Species Directional Comparison

The cross-species analysis restricted all three profiles to the same measured one-to-one ortholog universe. Human gene identifiers defined identical pathway memberships across human AKI, mouse disease, and mouse EGT rankings. We repeated complete-library fgsea and CAMERA analyses before and after cytosolic ribosomal-protein gene removal. A three-way association required concordant human-up and mouse-disease-up support plus EGT-down support at the analysis setting of 0.01. Spearman correlations summarize human–mouse disease concordance. The multiple-testing sensitivity analysis pooled Hallmark and Reactome probabilities into a combined BH family while keeping the enrichment criteria unchanged.

Human–mouse leading-edge overlap was evaluated within each pathway’s measurable common ortholog set using an upper-tail hypergeometric test. BH correction covered all eligible pathways in each collection and analysis mode. These overlap probabilities assess gene recurrence conditional on pathway membership. Random seeds were 202609061 for common-universe enrichment and 202609062 for annotation-marker removal.

### 2.7. Tissue Registration and Spatial Summaries

GSE171406 supplied one six-hour slice each for sham, IRI, and CLP from the source study’s 8–10-week-old 129/SvEv mice. Spatial assays provide tissue context with resolution and replication constraints [37]. In each slice, counts were normalized to 10,000 and log-transformed. Variable genes were standardized across spots; module scores were the mean of available standardized genes and were standardized again. At least three variable genes were required. Depth-adjusted scores were standardized residuals from a linear model containing log10 library size and log10 detected genes. Shared genes were removed from both modules in the disjoint analyses.

Eight frozen module pairs were summarized by Spearman correlation under raw or adjusted scoring and original or disjoint membership, giving 96 entries across three slices. A distance-trimmed, symmetric six-nearest-neighbor graph supplied spatial-lag summaries. Leave-one-occupied-block-out sensitivity used a fixed 4 × 4 partition; its ranges are not confidence intervals. Original high-resolution tissue images, barcodes, scale factors, and array coordinates were matched to all retained spots. Tissue colors were unchanged. Score overlays used opacity 0.8 and saturation at ±3 within-slice standard deviations. Appendix B provides all native views and overlays; Appendix A details registration and local spatial tests.

### 2.8. Visualization, Reproducibility, and Computational Assistance

Figures were generated in R using ggplot2, ComplexHeatmap, and igraph [38,39,40], with patchwork for panel assembly. For the seven-pathway bipartite network, an edge required the same gene to enter the leading edge of the same pathway in all three common-ortholog profiles after cytosolic ribosomal removal. All 99 genes and 174 edges were displayed; labels highlighted every gene with at least three shared pathway memberships. Edges encode annotation membership, not regulatory or protein interactions. The non-ribosomal display included every gene shared across at least two annotations after excluding both cytosolic and mitochondrial ribosomal proteins, including known mitochondrial ribosomal aliases. Gene effects and original contrast-specific BH FDR values were displayed without additional significance filtering. The leading-edge intersection display includes every nonempty intersection. Human Hallmark rows were clustered by Euclidean distance on full and ribosomal-removed NES values; EGT rows were ordered by measured-gene Jaccard distance. Both used complete linkage. Heatmaps preserve all 50 human Hallmark pathways, all 61 initially supported EGT pathways, and all 160 secondary PT-state combinations. Times New Roman at 16 pt was used in the original figure exports, with proportional reduction to the journal layout. Original figures and source data retain the specified font size.

For the human primary analysis, the gene-level volcano plot used the edgeR quasi-likelihood log2 fold change and BH-adjusted FDR from the seven-AKI versus three-control PT contrast. Points were classified as upregulated or downregulated when FDR *<* 0.05 and |log2 FC| ≥0.5; the remaining genes were shown as the reference distribution. Labels identify the most statistically prominent genes within the displayed significant set. This visualization is descriptive and does not replace the complete-library pathway tests.

Frozen rankings, gene sets, mappings, sample metadata, random seeds, complete testing families, and executable scripts were retained for reproduction and are available as described in the Data Availability Statement. Processing and annotation were interpreted in light of established single-cell analysis guidance [41]. OpenAI Codex assisted with language revision, code drafting, and figure refinement during manuscript preparation. The authors are responsible for checking the underlying data, executable analyses, references, and final interpretation. All authors reviewed and approved the final manuscript and take full responsibility for its content.

## 3. Results

### 3.1. Human Kidney Injury Defines a Coherent Inflammatory–Redox Program

Injury-associated human PT showed coordinated interferon, IL6–JAK–STAT3, and oxidative-stress transcription. These programs remained enriched after cytosolic ribosomal-protein genes were removed, indicating that the inflammatory and redox response extended beyond this component of the transcriptome (Figure 1). After removal, interferon-alpha and interferon-gamma responses, IL6–JAK–STAT3 signaling, and reactive oxygen species response had NES values of 2.25–2.91. The broader inflammatory-response set and KEAP1–NFE2L2 pathway also remained enriched (Table 2).

At the gene level, the same human contrast showed a broad distribution of injury-associated effects, with 300 genes meeting FDR *<* 0.05 and |log2 FC| ≥0.5. The most prominent changes included mitochondrial and redox-associated transcripts among the positive effects and stress-response transcripts among the negative effects (Figure 1c), providing gene-level context for the coordinated pathway signal.

The complete analysis tested 50 Hallmark and 1220 Reactome pathways and identified 187 enriched pathways under the concordant fgsea/CAMERA criterion. After removal of 80 cytosolic ribosomal-protein genes, 159 enriched pathways remained among 1254 testable pathways; 158 retained their original support and one additional pathway met the criterion. The complete 50-pathway Hallmark heatmap places the inflammatory and redox findings within the wider response (Figure A2 and Figure A3).

### 3.2. EGT Opposes Disease-Associated Mitochondrial and Stress Transcription

The intervention dataset allowed us to examine which disease-associated programs changed in the opposite direction during EGT treatment. In the cisplatin model, 61 of 1311 tested pathways showed disease-up/EGT-down enrichment with concordant support in both contrasts (Figure 2a). The response included nine Hallmark and 52 Reactome annotations. This comparison describes treatment-associated changes within the mouse study and complements the human injury findings.

Mitochondrial and stress-response associations remained after removal of the 56 ranked cytosolic ribosomal-protein genes. Twenty-five of 1304 testable pathways retained opposite-direction support: nine Hallmark and 16 Reactome pathways. All 25 had also been supported before removal. They included oxidative phosphorylation, reactive oxygen species response, mitochondrial protein import and translation, and DNA repair. These retained programs show that the EGT-associated response extends beyond cytosolic ribosomal transcription. Figure A4, Figure A5 and Figure A6 display every initially supported pathway across both contrasts and both analysis modes.

The gene-level display provides a complementary view of the treatment response. All 23,923 tested PT genes are shown with fitted EGT-versus-control log-fold changes in Figure 2b, with cytosolic ribosomal genes identified within the full distribution. The EGT-versus-control estimate is the arithmetic sum of the two contrast estimates, as defined in Equation (3); values nearer zero place the fitted estimate closer to the control reference. This view adds gene-level context to the coordinated pathway response, and the complete gene-level results are provided in Appendix A.4.

### 3.3. Cross-Species Concordance Narrows the Injury Response to an EGT-Associated Mitochondrial Core

To connect the injury and intervention analyses, we compared ranked statistics across unique one-to-one human–mouse orthologs. The matched table contained 9700 genes with complete human injury, mouse disease, and mouse EGT statistics. Human and mouse disease effects showed positive gene-level concordance (Spearman *ρ* = 0.242 for log-fold changes; *ρ* = 0.246 for ranked statistics), with 5757 genes sharing the same disease-effect sign. At the pathway level, 1212 common-universe pathways were testable in the full ranking and 1202 after ribosomal-gene removal. The full ranking yielded 31 human-up/mouse-disease-up/EGT-down pathways at the CAMERA analysis setting of 0.01, and a focused seven-pathway core persisted after removal (Figure 3 and Figure 2c,d). These retained associations comprised mTORC1 signaling, MYC targets V2, oxidative phosphorylation, complex I biogenesis, mitochondrial translation, mitochondrial translation elongation, and respiratory electron transport. Together, these results defined a mitochondrial bioenergetic–translation core represented by seven pathway annotations. The annotations overlapped in respiratory metabolism, mitochondrial protein synthesis, and broader biosynthetic responses rather than defining seven independent mechanisms. Table 3 summarizes the kidney-injury relevance and interpretation of each annotation.

### 3.4. Recurrent Highly Connected Genes Expand Apparent Pathway Breadth

The breadth of the EGT pathway response was partly explained by repeated inclusion of the same genes. Cytosolic ribosomal-protein genes accounted for only 53 of 954 unique disease-side leading-edge genes (5.6%), yet contributed 996 of 2788 pathway–gene memberships (35.7%). On the treatment side, 55 of 1223 unique genes (4.5%) supplied 1034 of 3513 memberships (29.4%; Figure 4). The complete intersection display retained all disease-side and treatment-side leading-edge unions, including sets retained after removal (Figure A7). This concentration explains why several pathway labels can describe a shared transcriptional component.

The seven-pathway core contained 99 genes entering the same pathway leading edge in all three common-ortholog profiles, contributing 174 pathway–gene memberships (Figure 2d). Under this stringent shared-edge rule, the five respiratory and mitochondrial translation annotations formed one connected component; mTORC1 and MYC targets V2 formed separate components. Of these genes, 64 were non-ribosomal and 35 encoded mitochondrial ribosomal proteins. Twenty-five non-ribosomal genes recurred in at least two annotations (Figure 4b,c). OXA1L recurred in five and NDUFAB1 in four; nine additional NDUF-family genes recurred in three. COX-family genes, UQCR10, UQCRB, UQCRQ, and TUFM each contributed to two shared leading edges. All 25 showed positive human and mouse disease effects and negative EGT effects. However, none met gene-level FDR *<* 0.05 in either mouse contrast, distinguishing coordinated pathway contributions from individually significant treatment effects. Supplementary Tables S3–S5 provide all gene effects, memberships, and pathway statistics.

Measured-gene overlap grouped the 61 EGT labels into 32 clusters at Jaccard similarity 0.25. The number ranged from 29 to 38 when the threshold varied from 0.15 to 0.35. Human enrichment also contained substantial overlap: 187 labels involved 3736 unique measured genes and 14,193 memberships, forming 81 clusters at the primary threshold. Their leading edges contained 1501 unique genes and 7636 memberships, with 966 genes appearing in more than one pathway. After ribosomal removal, the 159 enriched human labels formed 73 clusters. Thus, shared membership contributes to apparent pathway breadth in both settings, while the retained pathways still span multiple transcriptional programs.

### 3.5. Gene-Membership Controls Support the Contribution of Highly Connected Genes

Removing cytosolic ribosomal-protein genes had a larger effect on pathway retention than removing the same number of genes, including when ranking strength was matched (Figure 5). In the CAMERA-only control analysis, EGT retained 26 of 66 baseline opposite-direction pathways after ribosomal removal. The number-matched controls retained a median of 66 pathways (empirical 2.5th–97.5th percentiles, 64–66), and the signed-rank-matched controls retained 65 (61–66). Each control used 200 draws; none retained as few pathways as the ribosomal removal, giving *p*ref = 1/201 = 0.00498.

For human PT, 168 of 203 baseline CAMERA-enriched pathways remained after ribosomal removal. The corresponding medians were 201 (195–203) for number matching and 198 (184.95–202) for signed-rank matching. Again, all 200 draws retained more baseline pathways. These comparisons establish sensitivity to the composition of the removed group relative to the specified reference sets.

Annotation-degree matching further clarified the contribution of highly connected genes. Within the common-support subsets of 79 human and 25 EGT ribosomal genes, actual removal retained 176 human and 62 EGT baseline pathways, while matched reference draws retained medians of 161 (155–169) and 57 (57–58), respectively. Together with the number- and rank-matched controls, the human results supported annotation connectivity as a contributor to apparent pathway breadth. EGT matching covered only 25 of 56 targets and produced four distinct replacement sets across 1000 assignments; this control was exploratory. The conditional full-set analysis and complete reference distributions are reported in Appendix A.5.

Pooling Hallmark and Reactome into one BH family retained 192 enriched human pathways before removal and 165 afterward, together with 60 EGT opposite-direction pathways before removal and 24 afterward. The principal response categories were therefore represented under both correction schemes (Table A2).

### 3.6. Cellular States and Tissue Organization Provide Biological Context

The two contextual analyses placed the primary signatures within cellular states and tissue neighborhoods. The lineage dataset placed inflammatory programs within heterogeneous PT responses at four weeks. C14 showed inflammatory-response enrichment (NES 1.81; fgsea FDR 0.00409; CAMERA FDR 0.0383) across the published PT-state framework. The clustered, annotated heatmap in Figure A8 shows the complete 20-pathway-by-eight-state landscape and the location of this inflammatory state.

The spatial analysis registered all 5784 retained spots to their original tissue images (Figure 6). With disjoint modules and depth adjustment, injury–TNF/NF-kappaB correlations reached 0.630 in IRI and 0.368 in CLP, while injury–interferon-gamma correlations reached 0.186 and 0.139, respectively. The IRI injury–TNF association remained 0.580–0.655 under occupied-block omission. These spatial summaries place the principal inflammatory programs within registered tissue neighborhoods. Appendix B contains the native images and complete overlays, with local spatial-test results in Appendix A.8.

## 4. Discussion

This reanalysis identifies an EGT-associated transcriptional shift opposing a conserved mitochondrial injury program in proximal tubules. Seven overlapping pathway annotations retained human-up, mouse-disease-up, and EGT-down support after cytosolic ribosomal-protein gene removal. Their convergence is best interpreted as a mitochondrial bioenergetic–translation core, accompanied by broader growth and stress-response annotations. The human inflammatory–redox response provides the injury context, while recurrent gene membership explains part of the broader apparent EGT response.

The seven annotations represent connected biological functions rather than seven independent mechanisms. Oxidative phosphorylation describes ATP-generating metabolism, respiratory electron transport describes electron transfer, and complex I biogenesis describes assembly of a major entry point into the respiratory chain. Mitochondrial translation and its elongation subpathway supply mitochondrially encoded respiratory-chain subunits. The mTORC1 and MYC target annotations connect these processes to nutrient sensing, biosynthesis, and stress adaptation. Table 3 distinguishes the renal evidence for each annotation from the inference supported by this reanalysis.

Disrupted oxidative phosphorylation is a central feature of tubular energy dysfunction after injury, although its manifestations vary with model, cell state, and time. Adaptive kidney regeneration is associated with oxidative metabolism, while experimental sepsis can reduce complex I-dependent renal cortical respiration [8,42]. In an ischemia–reperfusion model, restoring the complex I subunit NDUFV1 improved mitochondrial homeostasis and attenuated renal injury [43]. These observations support the biological relevance of the respiratory annotations, but do not establish EGT action on an individual respiratory-chain component.

The positive injury NES in the present data should not be equated with increased respiratory capacity. Ranked transcriptional enrichment can coexist with biochemical dysfunction, compensatory gene expression, or differences in the sampled epithelial states. Likewise, EGT-associated negative enrichment indicates a relative transcriptional shift, not direct inhibition of respiration or demonstrated restoration of ATP production. The source EGT study reported renal and mitochondrial protection [16], and a separate cystinuria model linked EGT to glutathione metabolism and respiratory capacity [14]. These experimental findings motivate functional testing of the shared transcriptional core without replacing it.

The translation annotations connect respiratory-chain maintenance with protein synthesis. Pathogenic MRPS7 variation impairs mitochondrial translation and respiratory-chain function and can cause progressive renal failure [44]. This inherited mitochondrial disease provides renal plausibility, rather than direct evidence for an acquired AKI or EGT mechanism. Experimental renal tubular injury also links mitochondrial RNA dysregulation to PKR–eIF2-alpha stress signaling [47]. Oxidative ribosome damage and translational quality control offer additional biological context [12,48]. The elongation annotation is nested within mitochondrial translation and should not be counted as a separate mechanism.

The broader mTORC1 and MYC annotations require particularly careful interpretation. Post-ischemic nutrient restriction can suppress mTORC1 and worsen tubular injury and fibrosis, demonstrating that reduced mTORC1 activity is not uniformly protective [45]. Experimental work also connects c-MYC regulation to mitochondrial ATP handling, oxidative stress, and mitophagy in AKI [46]. However, enrichment of MYC targets V2 does not measure MYC occupancy or establish MYC-dependent mediation of the EGT response. The present results support coordinated biosynthetic transcription, with biochemical activity and causal direction remaining unresolved.

Gene-level decomposition makes the conserved core more useful for follow-up. OXA1L and NDUFAB1 recurred in five and four pathway leading edges, respectively, across all three profiles. Additional shared contributors included NDUF-family, COX-family, UQCR-family, and TUFM genes. Their membership pattern links respiratory-chain assembly and electron transport with mitochondrial translation. These are candidates prioritized by pathway contribution, rather than validated EGT targets. None of the 25 recurrent non-ribosomal candidates met gene-level FDR *<* 0.05 in either mouse contrast; their contribution emerges from coordinated ranking across gene sets.

The human injury program is consistent with epithelial stress in the source AKI study [20]. Interferon, IL6–JAK–STAT3, reactive oxygen species, and KEAP1–NFE2L2 annotations remained represented after annotation-marker and cytosolic ribosomal-gene removal. This retention links the mitochondrial EGT-associated core to an injury environment involving immune signaling and redox adaptation. The two programs need not arise in identical cell states or imply the same direction of biochemical change.

Pathway membership remains an essential interpretive layer. Cytosolic ribosomal-protein genes represented 5.6% of unique disease-side leading-edge genes but 35.7% of memberships; treatment-side values were 4.5% and 29.4%, respectively. Together, the matching analyses indicate that annotation connectivity materially contributes to repeated pathway representation. This inference was strongest for the human analysis; the EGT degree-matched control had a more restricted feasible matching space and should therefore be regarded as exploratory. Only 25 of 56 EGT ribosomal targets had common support, and 1000 assignments yielded four distinct replacement sets. The narrow reference distribution therefore does not represent 1000 independent biological observations or establish a ribosome-specific causal effect.

The PT-state and spatial analyses supply cellular and anatomical context. Inflammatory-response enrichment was observed in C14 within the published PT-state framework. This observation is consistent with heterogeneous repair responses in the lineage study [21], but does not establish a C14-specific treatment response. In registered tissue sections, injury and TNF/NF-kappaB scores covaried most strongly in IRI tissue after depth adjustment. Each spatial condition contributed one section, limiting inference to descriptive within-section associations.

Several limitations define the scope of the proposed core. Human severe AKI and the mouse cisplatin intervention differ in injury context, sampling, and cellular composition. The mouse analysis contained three libraries per group, and the contextual datasets did not independently replicate EGT effects. Common ortholog restriction improved comparability while excluding species-specific or unmapped genes. The seven annotations were defined at the specified CAMERA correlation and multiple-testing settings; removal of cytosolic ribosomal genes also left mitochondrial ribosomal contributions intact. These analyses neither demonstrate seven independent mechanisms nor prove treatment-mediated biochemical rescue.

The results prioritize a coordinated experimental evaluation of EGT during tubular injury and recovery. Respiratory flux, ATP generation, complex I assembly, mitochondrial translation, and oxidative damage should be measured alongside tubular function. Perturbing recurrent contributors such as OXA1L, NDUFAB1, or TUFM could test whether they mediate, accompany, or compensate for the EGT-associated transcriptional shift. Independent validation would determine whether this conserved gene-set pattern marks functional recovery and supports further therapeutic investigation.

## 5. Conclusions

EGT-associated transcription opposed a conserved mitochondrial injury program in human and mouse proximal tubules. Seven overlapping annotations retained three-way pathway support after cytosolic ribosomal-protein gene removal, defining a mitochondrial bioenergetic–translation core. Recurrent non-ribosomal contributors included OXA1L, NDUFAB1, respiratory-chain genes, and TUFM, providing candidates for functional evaluation. Gene-membership controls also showed how highly connected genes broaden apparent pathway enrichment, with the restricted EGT degree-matched analysis remaining exploratory. These results prioritize coordinated mitochondrial endpoints for EGT studies without establishing causal targets or biochemical rescue.

## Figures and Tables

**Figure 1:**
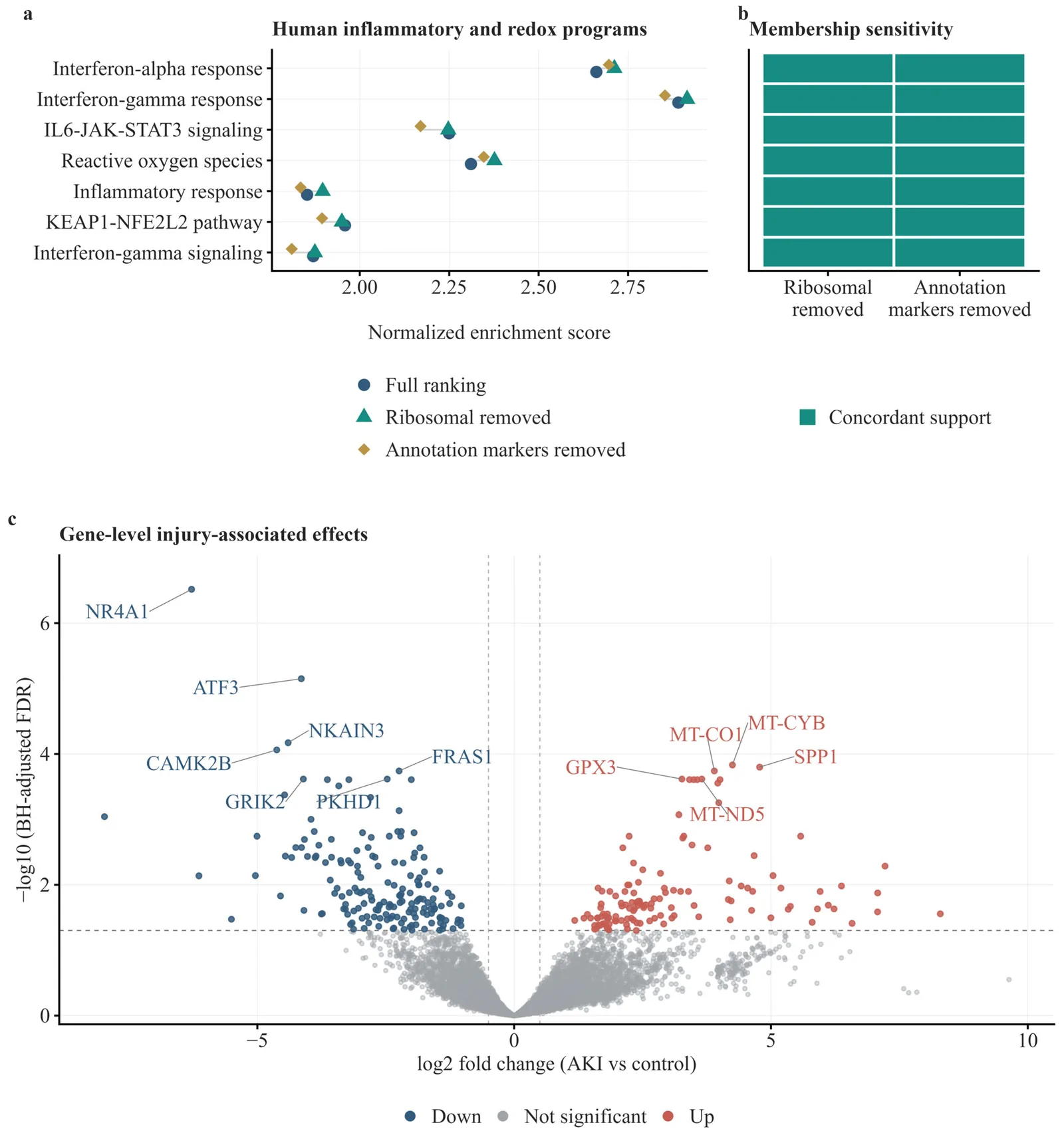
Inflammatory and redox enrichment in injured human proximal tubules. (**a**) Normalized enrichment scores for seven inflammatory and redox programs in the full ranking, after cytosolic ribosomal-protein gene removal, and after removal of the six annotation markers. Points indicate concordant fgsea/CAMERA support under the analysis setting described in Materials and Methods. (**b**) Pathway-specific support after cytosolic ribosomal-protein removal and after direct annotation-marker removal. (**c**) Gene-level volcano plot for the human AKI-versus-control PT contrast. Coral and blue points meet FDR *<* 0.05 with |log2 FC| ≥ 0.5 and indicate positive and negative effects, respectively; gray points show the remaining genes. The dashed vertical lines indicate the log2 FC thresholds of −0.5 and 0.5, and the dashed horizontal line indicates the FDR threshold of 0.05. Human contrast: seven AKI and three nephrectomy-control PT pseudobulks. Source: GSE210622.

**Figure 2:**
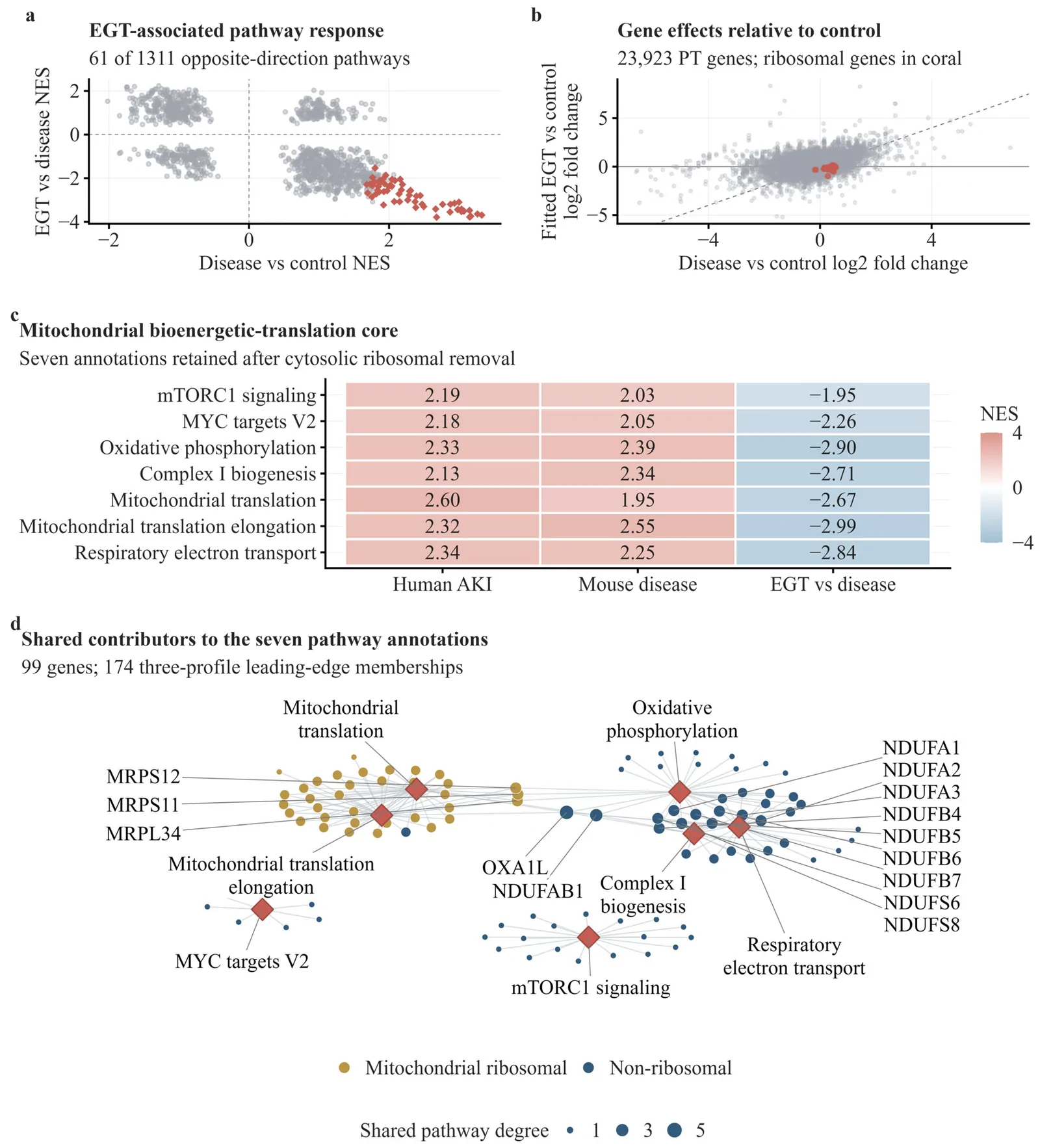
EGT-associated transcription, the seven-pathway core, and shared gene contributors. (**a**) All 1311 mouse pathway comparisons; coral diamonds mark 61 disease-up/EGT-down annotations. (**b**) Disease effects and fitted EGT-versus-control estimates for all 23,923 PT genes (Equation (3)); coral marks cytosolic ribosomal genes. The diagonal denotes no treatment-associated change; horizontal zero is the fitted control reference. (**c**) Normalized enrichment scores (NES) for seven annotations retaining human-up/mouse-disease-up/EGT-down support after cytosolic ribosomal removal. Every cell meets concordant fgsea/CAMERA FDR *<* 0.05 at CAMERA correlation 0.01. (**d**) These annotations (diamonds) and all 99 genes entering the same pathway leading edge in all three profiles; the 174 edges encode membership, not regulation. Blue and gold indicate non-ribosomal and mitochondrial ribosomal genes. Node size indicates shared pathway degree; all 14 genes with degree ≥ 3 are labeled. The common-universe selection is detailed in Section 3.3. Sources: GSE329941 and GSE210622.

**Figure 3:**
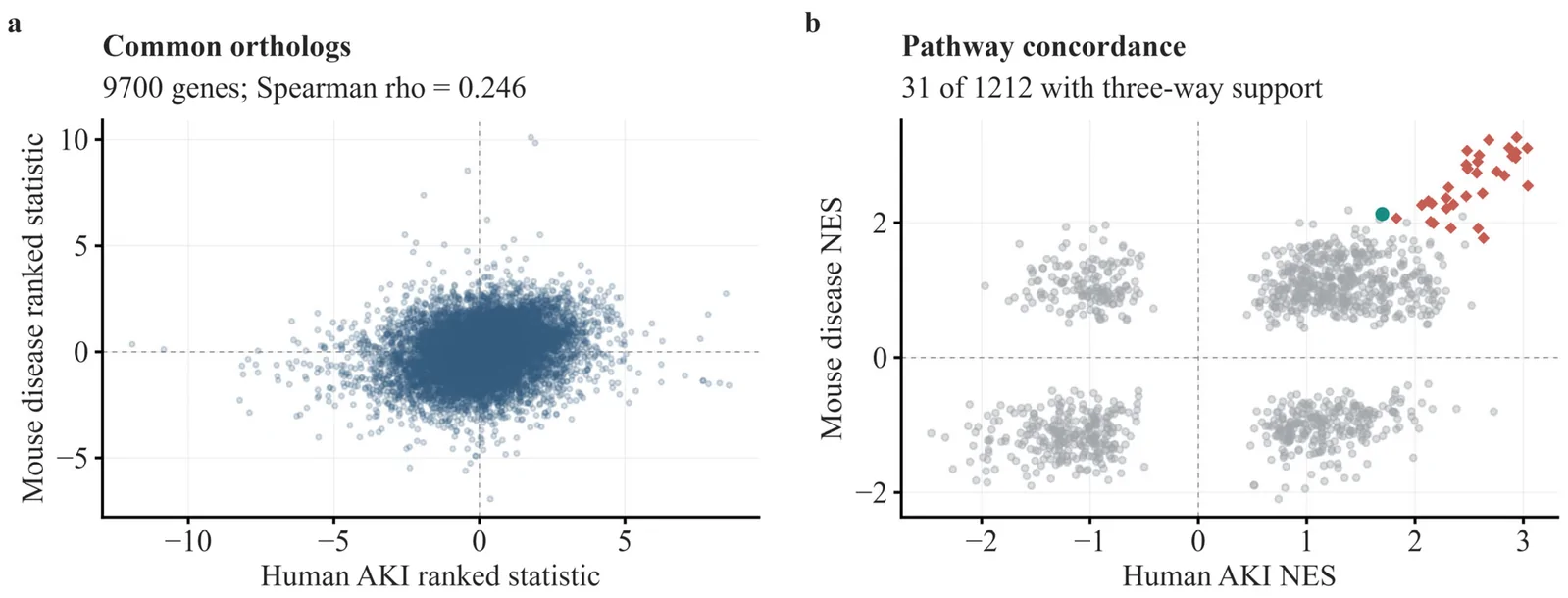
Cross-species concordance connects human injury with the mouse intervention model. (**a**) Human AKI and mouse disease ranked statistics for all 9700 measured one-to-one orthologs. (**b**) Human and mouse disease normalized enrichment scores (NES) for all 1212 jointly testable pathways. Coral diamonds indicate the 31 pathways with human-up/mouse-disease-up/EGT-down three-way support, teal points indicate the remaining jointly disease-up pathway(s), and gray points represent the other jointly testable pathways that did not meet the three-way support criterion. Seven annotations retain three-way support after cytosolic ribosomal removal, among 1202 testable pathways; their scores and shared contributors are assembled in Figure 2c,d. Additional sensitivity results appear in Appendix A.3. Sources: GSE210622, GSE329941, and the retained one-to-one ortholog table.

**Figure 4:**
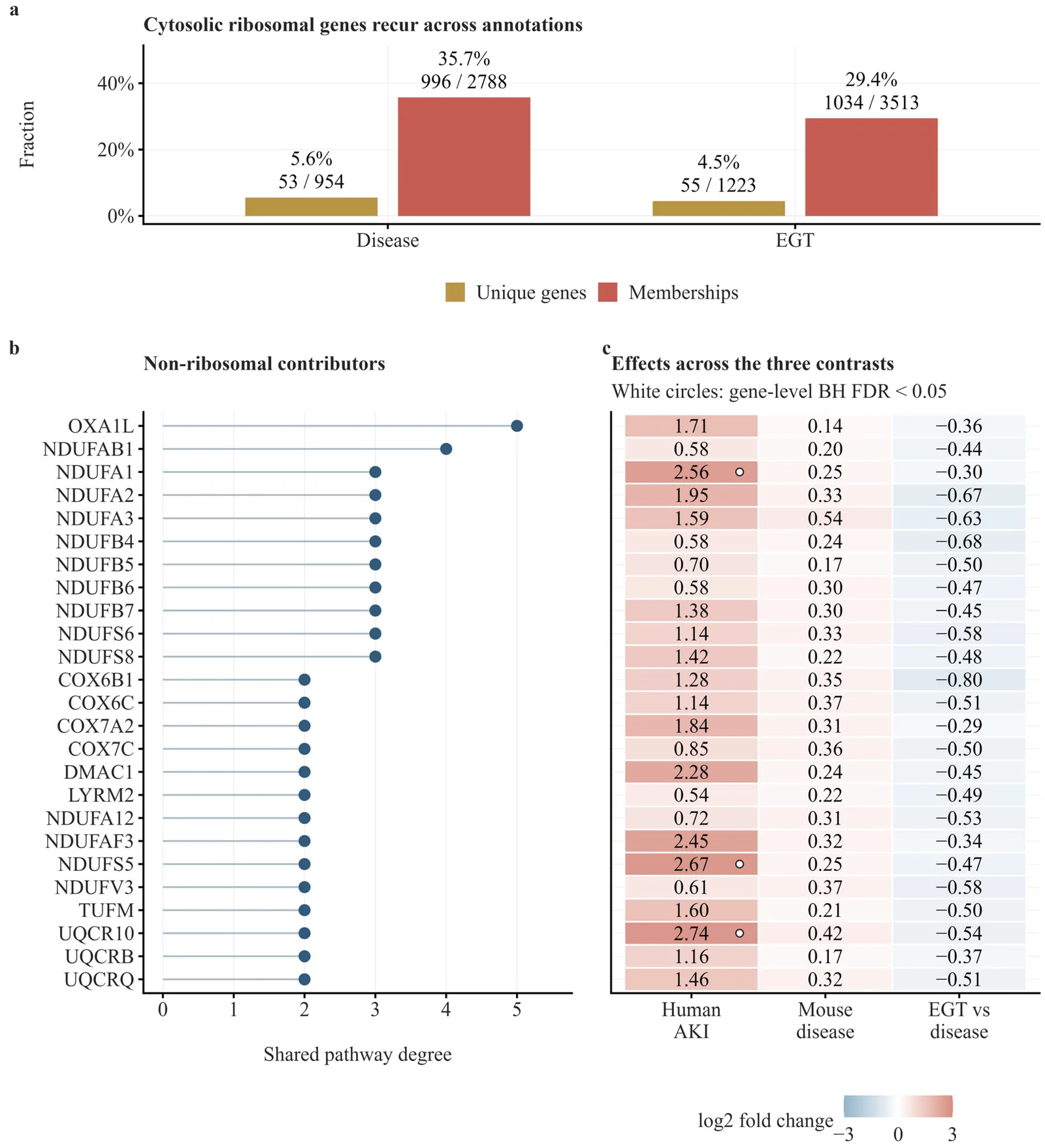
Ribosomal membership concentration and recurrent non-ribosomal contributors. (**a**) Cytosolic ribosomal fractions of unique genes and total memberships across the 61 initial EGT-associated annotations, with explicit denominators. (**b**) All 25 non-ribosomal genes entering the same pathway leading edge in all three common-ortholog profiles for at least two of the seven retained annotations. Degree counts these shared memberships; both cytosolic and mitochondrial ribosomal proteins were excluded. (**c**) Original gene-level log2 fold changes for the same genes and order. White circles mark contrast-specific BH FDR *<* 0.05; no displayed gene meets this criterion in either mouse contrast. Selection reflects pathway contribution rather than individual differential significance or causal regulation. Complete membership accounting appears in Figure A7. Sources: GSE210622 and GSE329941.

**Figure 5:**
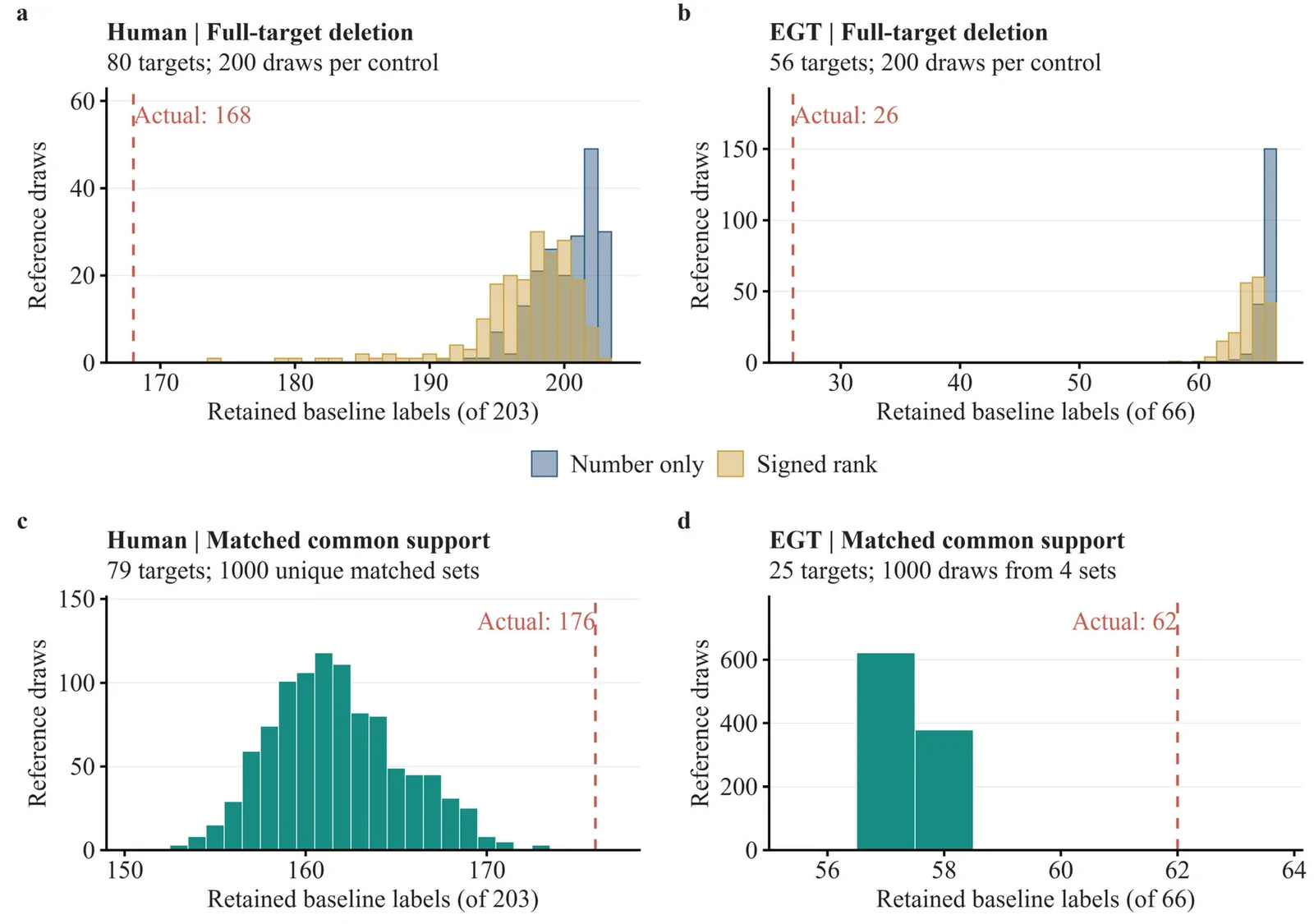
Gene-membership controls for pathway retention. (**a**,**b**) Retained baseline pathway counts after removal of all 80 human or 56 mouse ribosomal targets, compared with 200 number-matched and 200 signed-rank-matched non-ribosomal draws. Dashed coral lines mark actual removal (168 human; 26 EGT). (**c**) Degree-and-rank matching within the human common-support subset of 79 genes. (**d**) The EGT common-support result across 1000 matched assignments. Actual subset deletions retain 176 and 62 pathways. All panels use CAMERA-only baseline retention with an assumed inter-gene correlation of 0.01 and full-library BH correction. Matching details and complete numerical results are given in Appendix A.5. Sources: GSE210622 and GSE329941.

**Figure 6:**
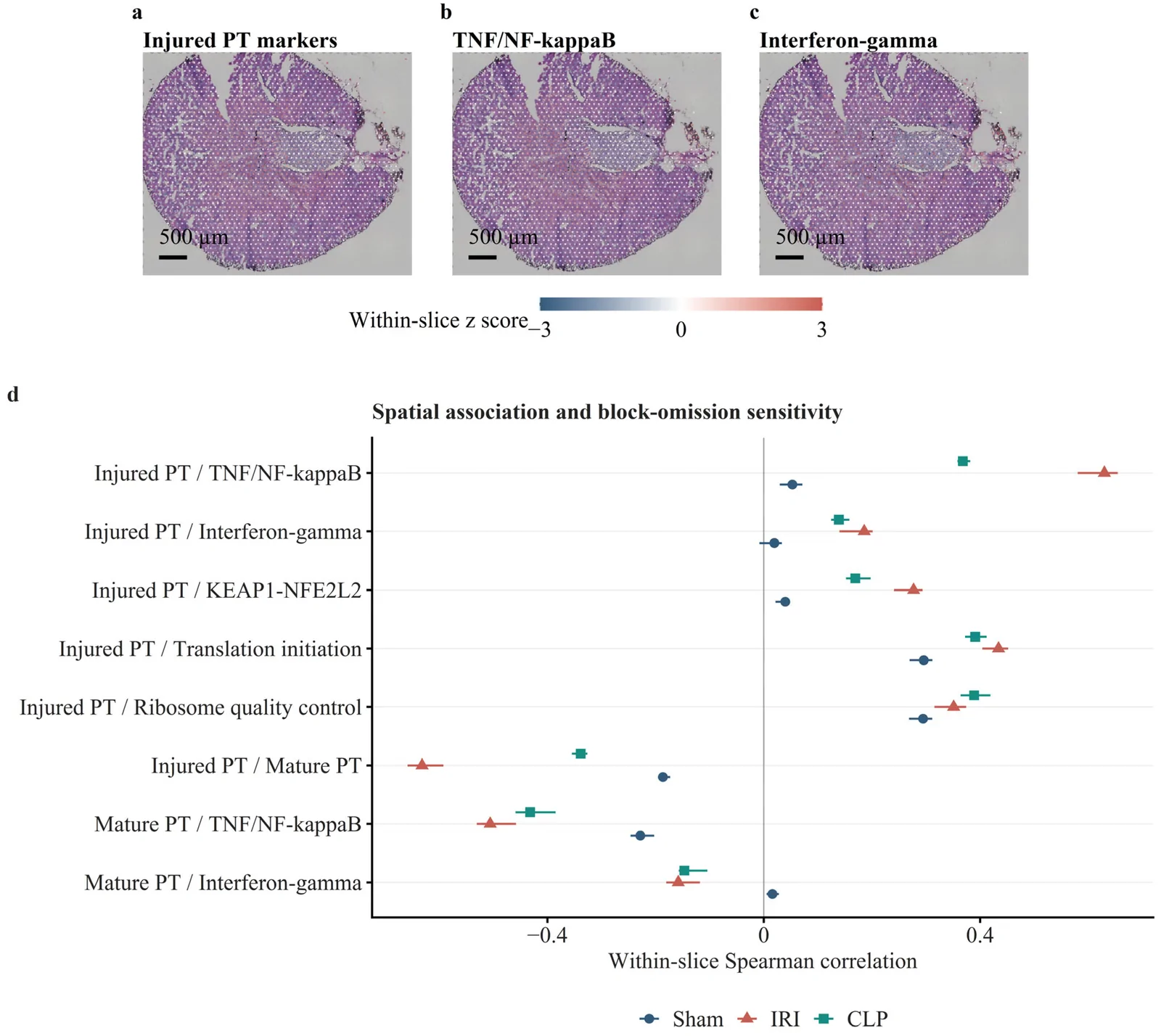
Tissue organization of injury-associated and inflammatory transcription. (**a**–**c**) Registered IRI tissue with injured-PT, TNF/NF-kappaB, and interferon-gamma scores on all 2063 retained spots. Scores are depth-adjusted and standardized within the slice; inflammatory overlays exclude shared injury-module genes. The same crop contains all retained spots in each panel. Scale bars: nominal 500 µm, derived from the supplied image scale and 55 µm capture-spot specification. (**d**) Spearman correlations for all eight disjoint module pairs in sham, IRI, and CLP. Segments show occupied-block omission minima and maxima, not confidence intervals. One slice per condition contributes 1833, 2063, and 1888 spots, respectively. Scores saturate at ±3 within-slice standard deviations with opacity 0.8; equal colors across slices do not imply equal absolute expression. Full native fields appear in Appendix B. Source: GSE171406.

**Table 1:** Public datasets and focal analysis units. PT, proximal tubule; GFP, green fluorescent protein; IRI, ischemia–reperfusion injury; CLP, cecal ligation and puncture.

GEO Accession	Samples in the Focal Analysis	Analysis Tier and Role
GSE210622 [20]	Seven AKI and three nephrectomy-control PT pseudobulks	Primary analysis: human AKI-associated transcription
GSE329941 [16]	Three libraries each from control, AKI–CKD, and AKI–CKD with EGT	Primary analysis: disease and treatment contrasts within a cisplatin model
GSE209610 [21]	Three GFP-positive and two control samples at four weeks; eight PT states	Contextual analysis: responses within existing RNA-defined states
GSE171406 [22]	One six-hour slice each for sham, IRI, and CLP; 1833, 2063, and 1888 spots	Contextual analysis: descriptive localization within individual tissue sections

**Table 2:** Selected inflammatory and redox programs in human PT after cytosolic ribosomal-protein gene removal. Adjusted probabilities use each complete pathway library; CAMERA assumes inter-gene correlation 0.01. NES, normalized enrichment score; FDR, false-discovery rate.

Program	NES	fgsea FDR	CAMERA FDR
Hallmark interferon-alpha response	2.712	5.83 × 10^−14^	2.84 × 10^−11^
Hallmark interferon-gamma response	2.915	4.96 × 10^−23^	1.99 × 10^−12^
Hallmark IL6–JAK–STAT3 signaling	2.247	4.34 × 10^−6^	8.58 × 10^−6^
Hallmark reactive oxygen species	2.376	8.53 × 10^−7^	3.84 × 10^−5^
Hallmark inflammatory response	1.896	0.000122	0.002745
Reactome interferon-gamma signaling	1.874	0.002045	0.003080
Reactome KEAP1–NFE2L2 pathway	1.950	0.000211	0.004902

**Table 3:** Renal relevance and interpretation of the seven retained pathway annotations. All seven met human-up/mouse-disease-up/EGT-down criteria after cytosolic ribosomal-protein gene removal. The literature establishes biological context; it does not validate EGT mediation of each annotation.

Annotation	Renal Relevance and Interpretive Boundary
Oxidative phosphorylation	ATP-generating metabolism is linked to adaptive tubular regeneration; impaired oxidative metabolism accompanies injury [8]. Positive NES does not measure ATP output.
Respiratory electron transport	Experimental septic AKI alters cortical electron transport and reduces complex I-dependent respiration [42]. This annotation overlaps oxidative phosphorylation.
Complex I biogenesis	Restoring the complex I subunit NDUFV1 attenuated ischemic renal injury and improved mitochondrial homeostasis [43]. The annotation identifies assembly-related transcription, not measured complex I activity.
Mitochondrial translation	MRPS7-associated translation defects cause respiratory-chain dysfunction and can include renal failure [44]. This is inherited-disease evidence, not direct evidence of an EGT mechanism in AKI.
Mitochondrial translation elongation	Elongation is a component of mitochondrial protein synthesis required for respiratory-chain maintenance. The renal relevance follows the translation evidence above [44]; it is a nested annotation, not an independently validated injury mechanism.
mTORC1 signaling	Nutrient sensing and autophagy influence post-ischemic repair; mTORC1 suppression can worsen injury under caloric restriction [45]. Transcript enrichment does not establish kinase activity or a uniformly protective direction.
MYC targets V2	Experimental c-MYC regulation connects mitochondrial ATP handling, oxidative stress, and mitophagy in AKI [46]. This broad target set does not establish MYC activation or direct EGT targeting.

**Figure A1:**
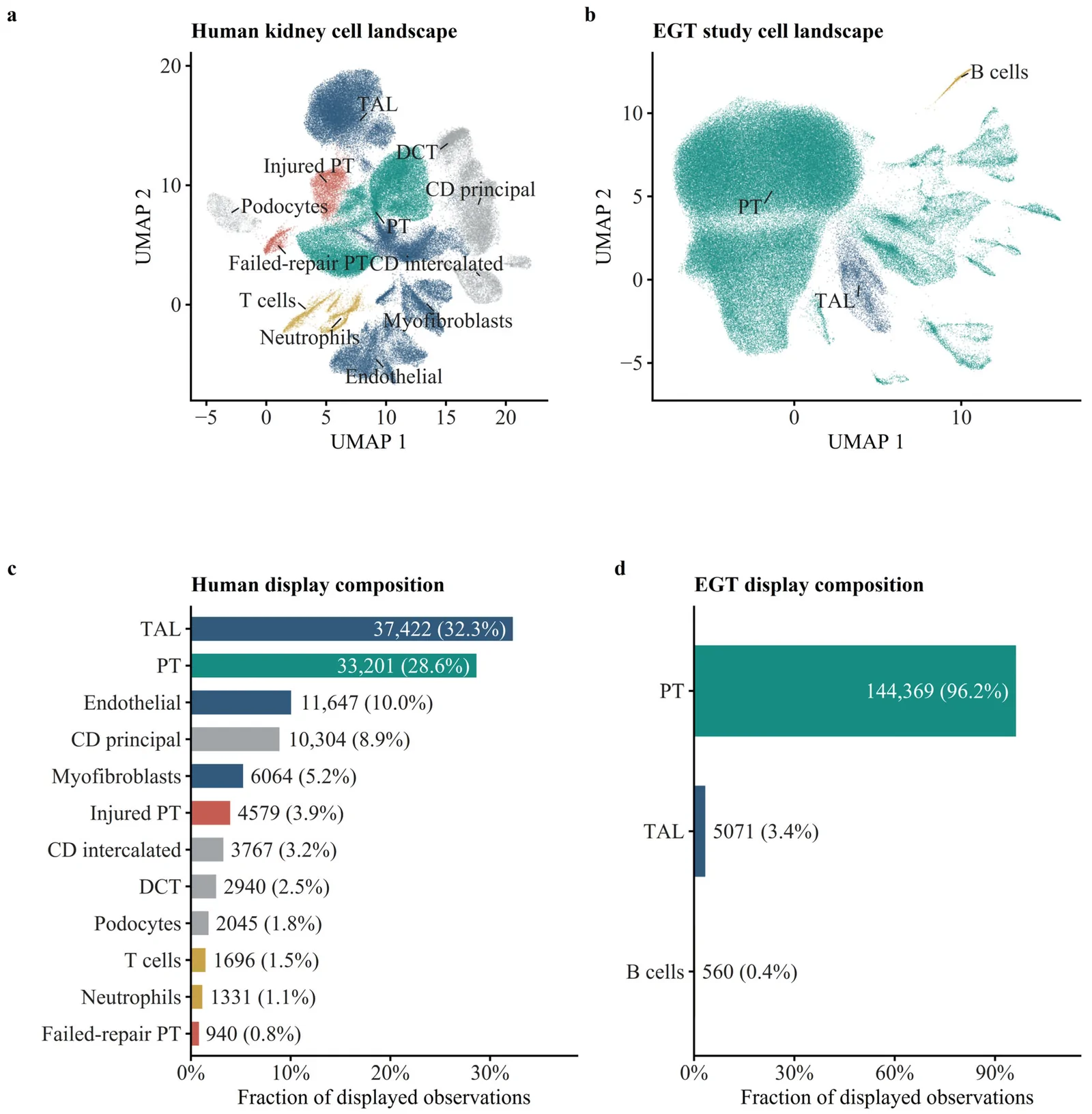
Existing cell landscapes and displayed annotation composition. (**a**,**b**) Existing UMAP coordinates with inherited marker-based annotations for human GSE210622 and mouse EGT GSE329941. Human observations span the original 14-library reference; the EGT coordinates are an inherited stratified display sample. (**c**,**d**) Counts and fractions of displayed observations in each annotation category. The human export contains 12 categories and the EGT export PT, thick ascending limb (TAL), and B-cell categories. Labels are provisional, and sample/group identifiers are absent from these display tables. These panels provide annotation context without inferring treatment-group abundance differences, new cell identities, or state transitions. Focal sample-level contrasts are specified in Table 1.

**Figure A2:**
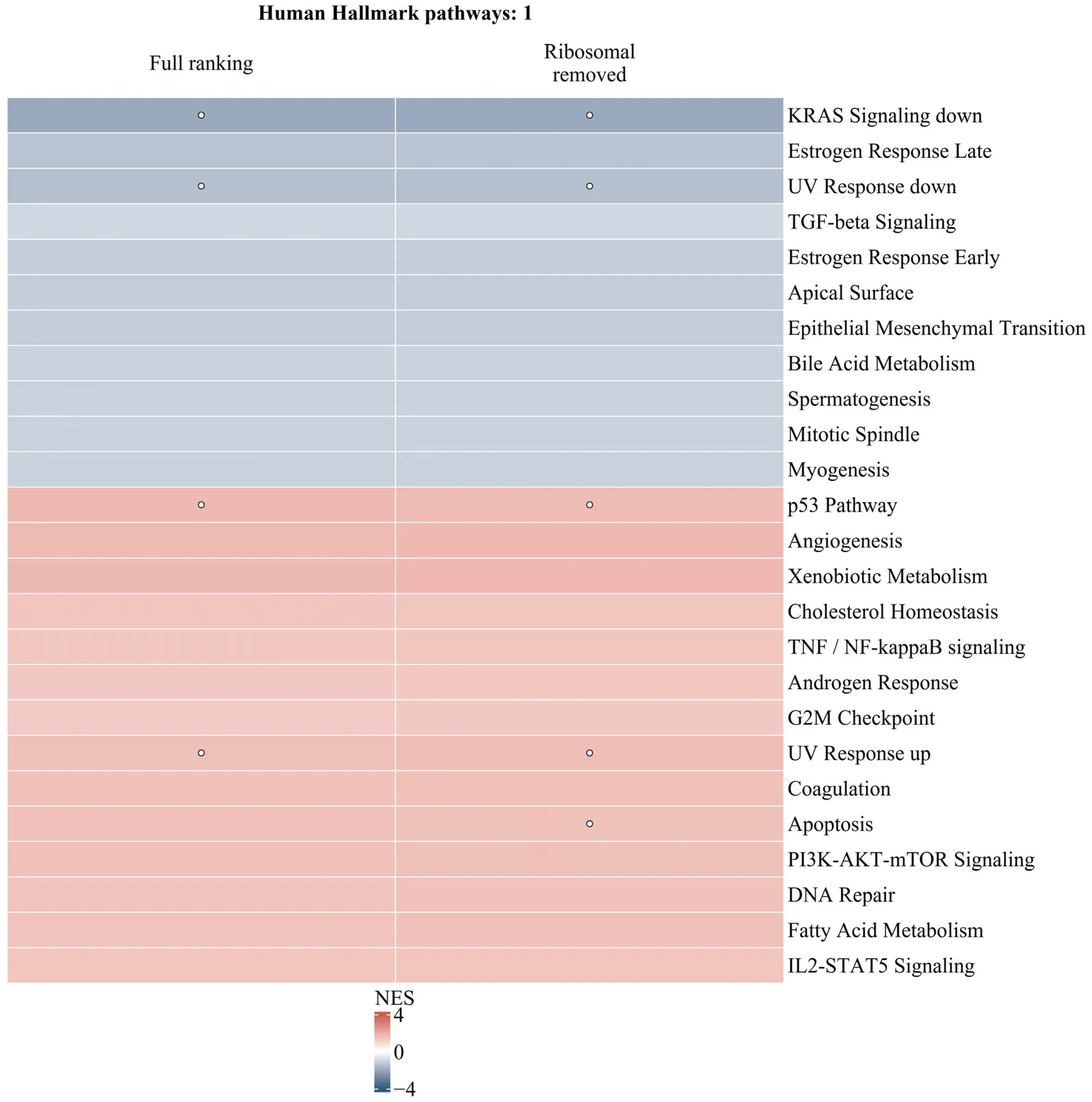
Complete human Hallmark landscape, part 1. The first 25 pathways in the full 50-pathway clustering order. Columns give NES before and after cytosolic ribosomal-protein gene removal. White-centered circles mark concordant fgsea/CAMERA FDR *<* 0.05 within each original complete testing family, with CAMERA correlation 0.01. Rows use complete-linkage clustering of Euclidean NES distances. Colors share the −4 to +4 scale in Figure A3. Source: GSE210622.

**Figure A3:**
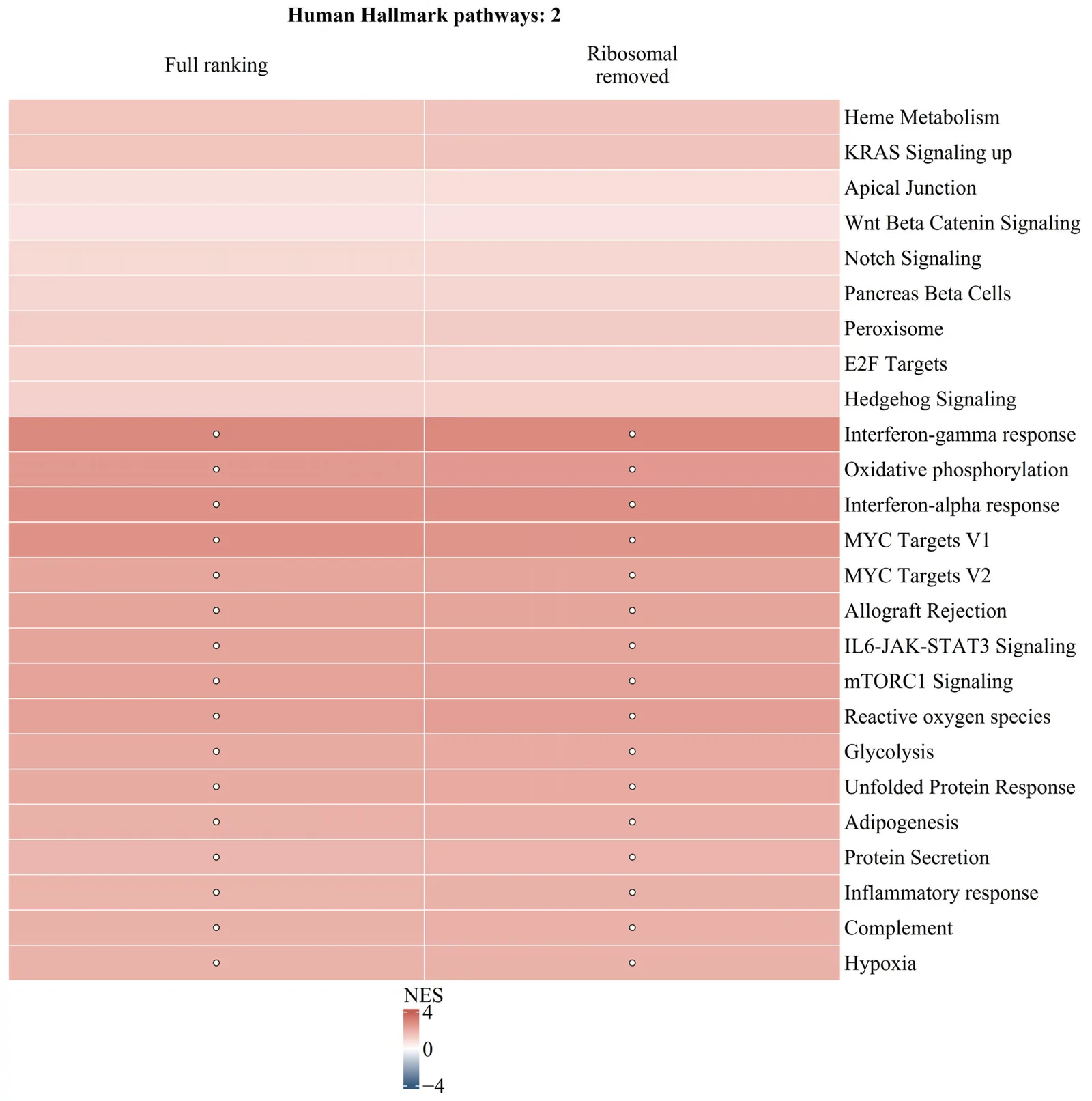
Complete human Hallmark landscape, part 2. The remaining 25 pathways in the same clustering order as Figure A2. Colors, columns, and support markers follow that figure; no pathway is omitted from the combined display. The heatmap places the principal inflammatory and redox findings within all tested Hallmark pathways. Source: GSE210622.

**Figure A4:**
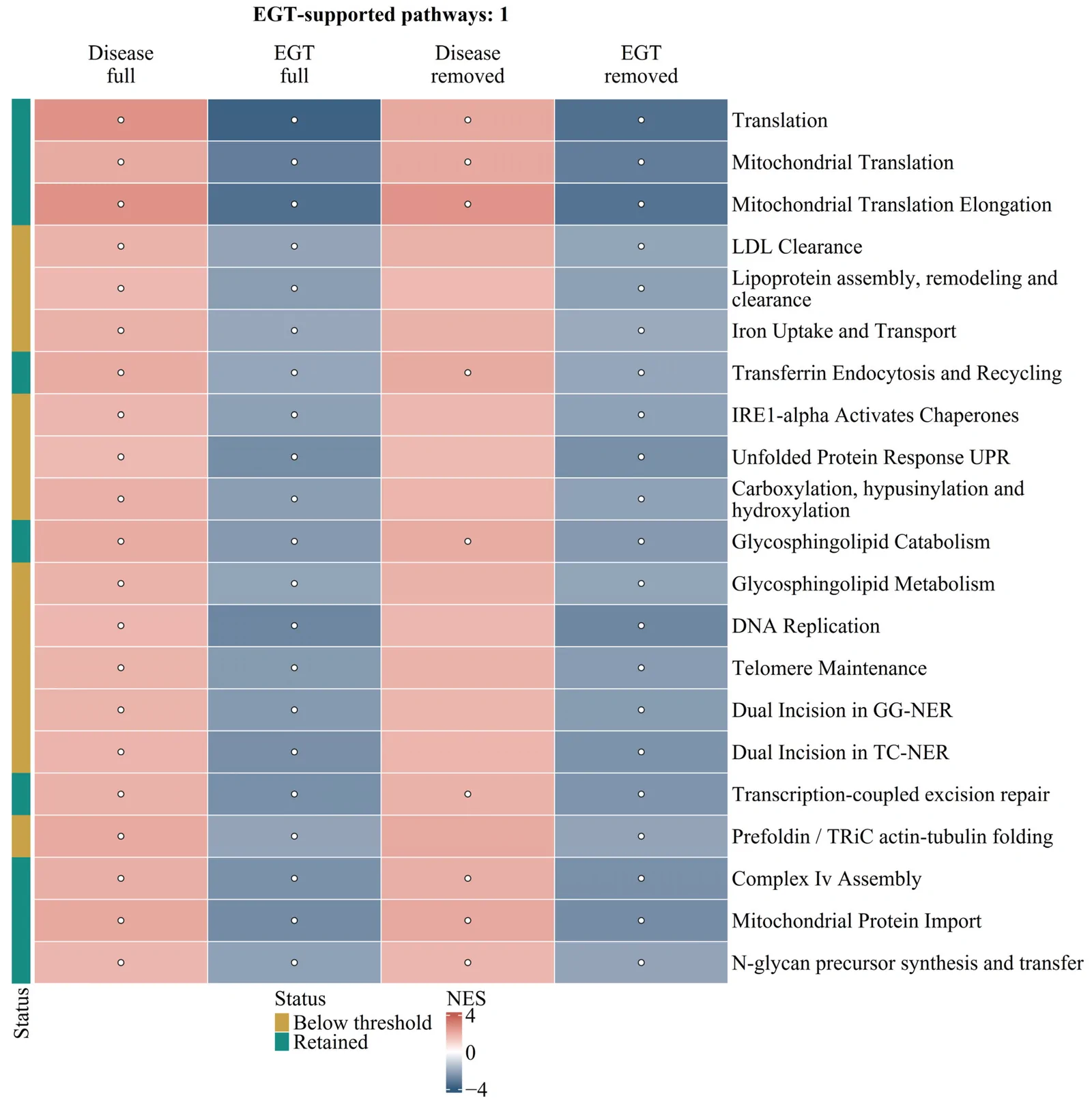
EGT-associated pathways, part 1. The first 21 of 61 initially supported pathways, ordered by complete-linkage clustering of measured-gene Jaccard distance. Columns give disease-versus-control and EGT-versus-disease NES before and after cytosolic ribosomal-protein gene removal. The row annotation identifies retained dual-contrast support. Circles mark concordant fgsea/CAMERA FDR *<* 0.05 in each individual contrast at correlation 0.01; gray cells are non-testable. Correction uses the full Hallmark or Reactome library. Viral-process labels refer to overlapping host-gene annotations and do not imply infection. Sources: GSE329941 and MSigDB/Reactome.

**Figure A5:**
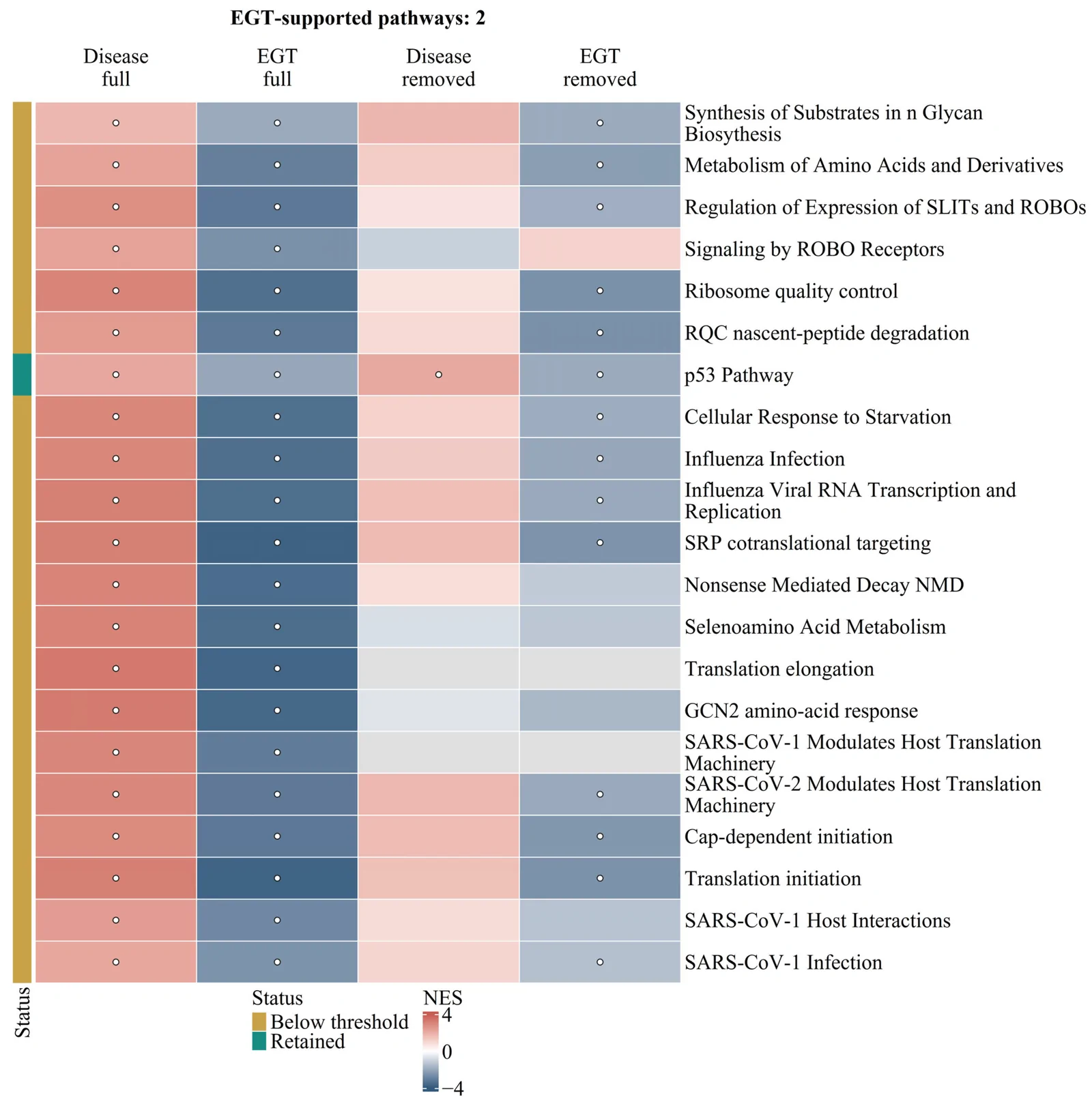
EGT-associated pathways, part 2. Pathways 22–42 from the same 61-pathway order. Columns, row annotation, support markers, and color scale follow Figure A4. Pagination preserves every initially supported pathway. Source: GSE329941.

**Figure A6:**
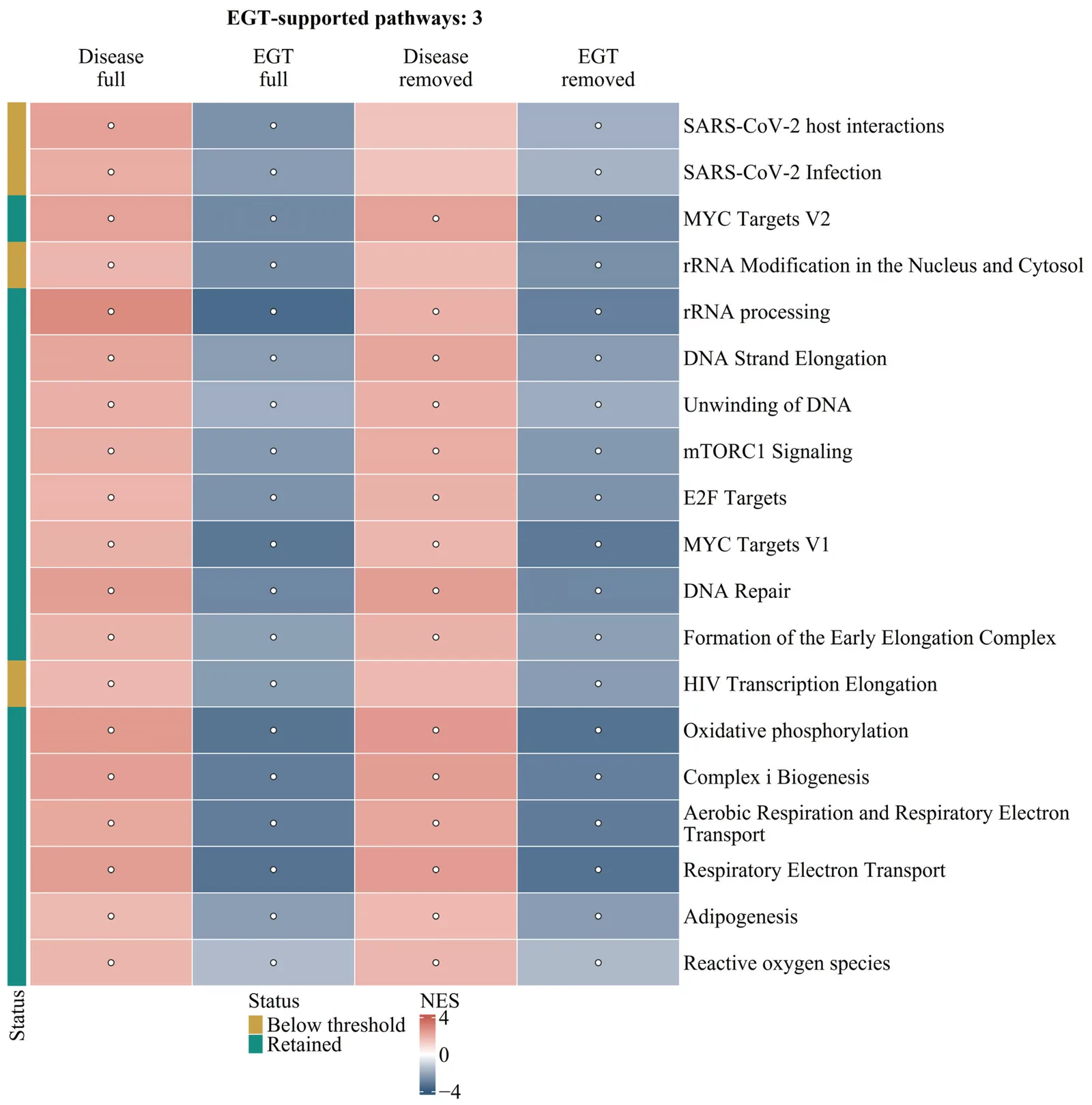
EGT-associated pathways, part 3. Pathways 43–61 from the same order. Columns, annotation, and support markers follow Figure A4. The complete display shows both the ribosomal-removal-sensitive annotations and retained mitochondrial or stress-response associations. Source: GSE329941.

**Figure A7:**
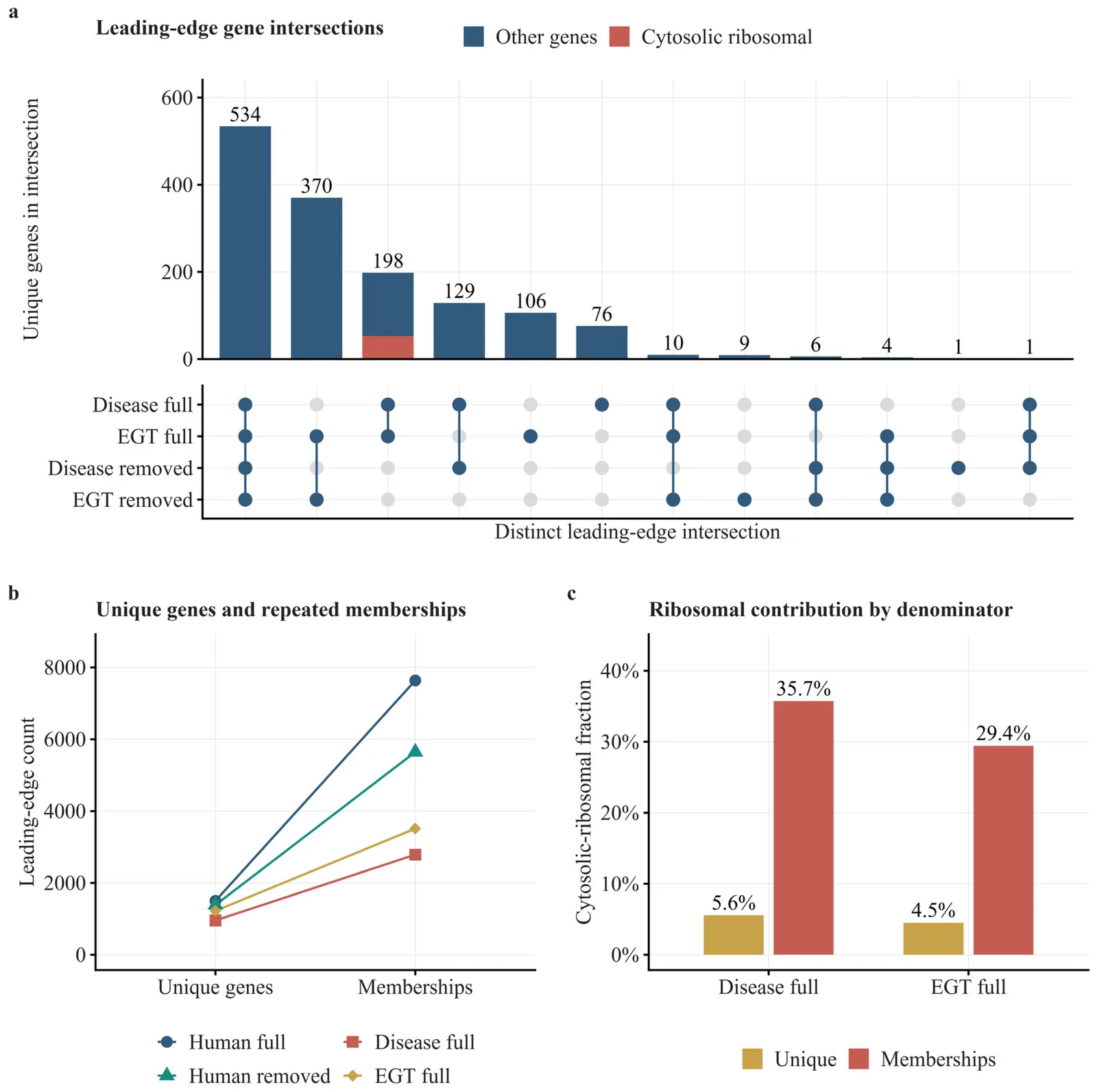
Complete leading-edge intersections and membership accounting. (**a**) Exact intersections among disease-side and treatment-side gene unions, before and after cytosolic ribosomal removal. (**b**) Unique genes and repeated pathway–gene memberships. (**c**) Cytosolic ribosomal fractions under both denominators. A gene contributes one membership for each pathway in which it occurs. Sources: GSE210622 and GSE329941.

**Figure A8:**
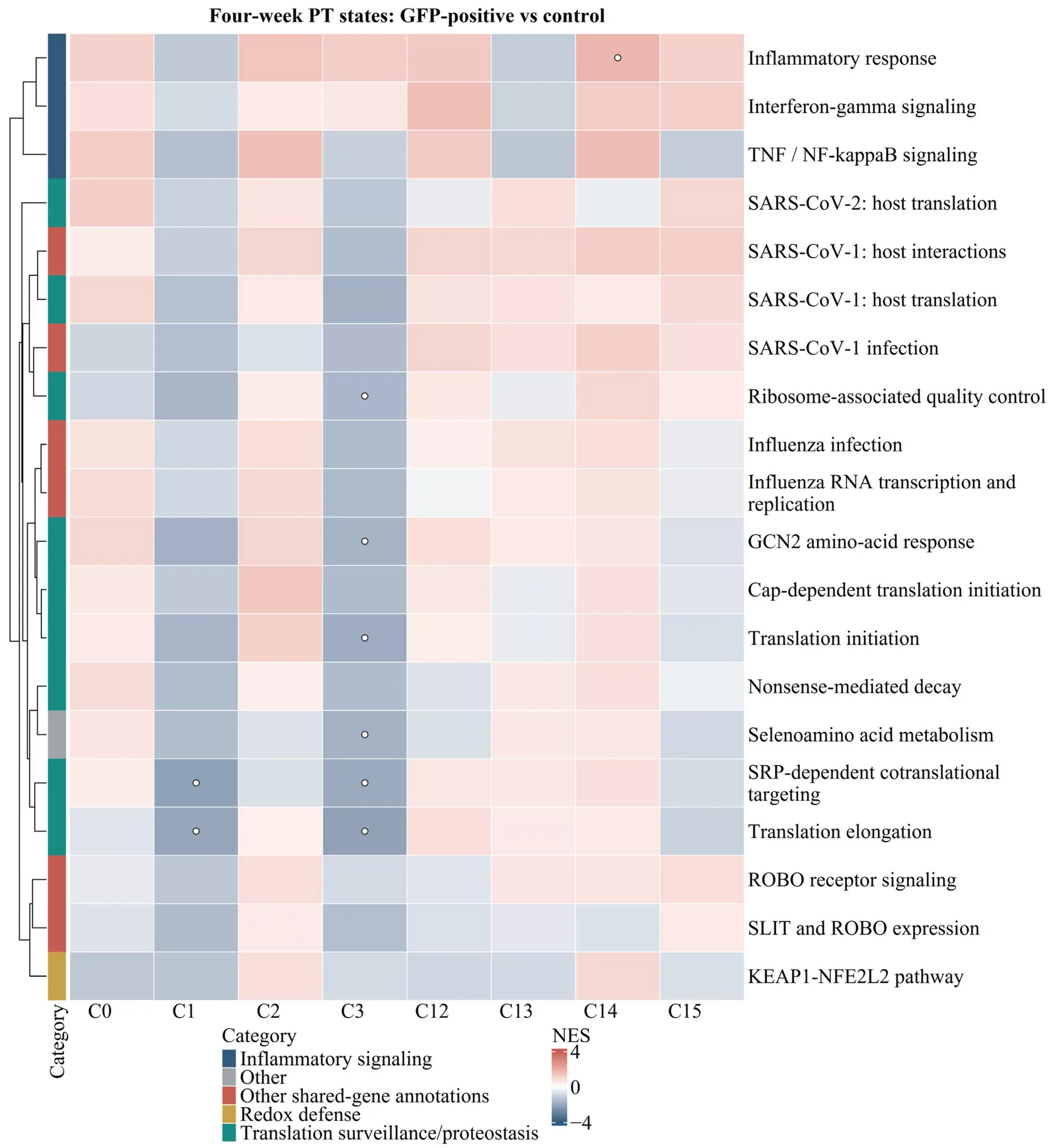
Clustered and annotated PT-state pathway landscape. All 20 pathways across eight existing PT states from four-week GFP-positive versus control contrasts are shown. Rows use complete-linkage Euclidean clustering of NES profiles; the side annotation groups related pathway labels. Circles require matching-direction fgsea and CAMERA results after separate correction across all 160 tests for each method. The inflammatory-response signal in C14 is highlighted within the complete state landscape. Database viral labels describe shared host-gene annotations. There are three GFP-positive and two control samples. Source: GSE209610.

**Figure A9:**
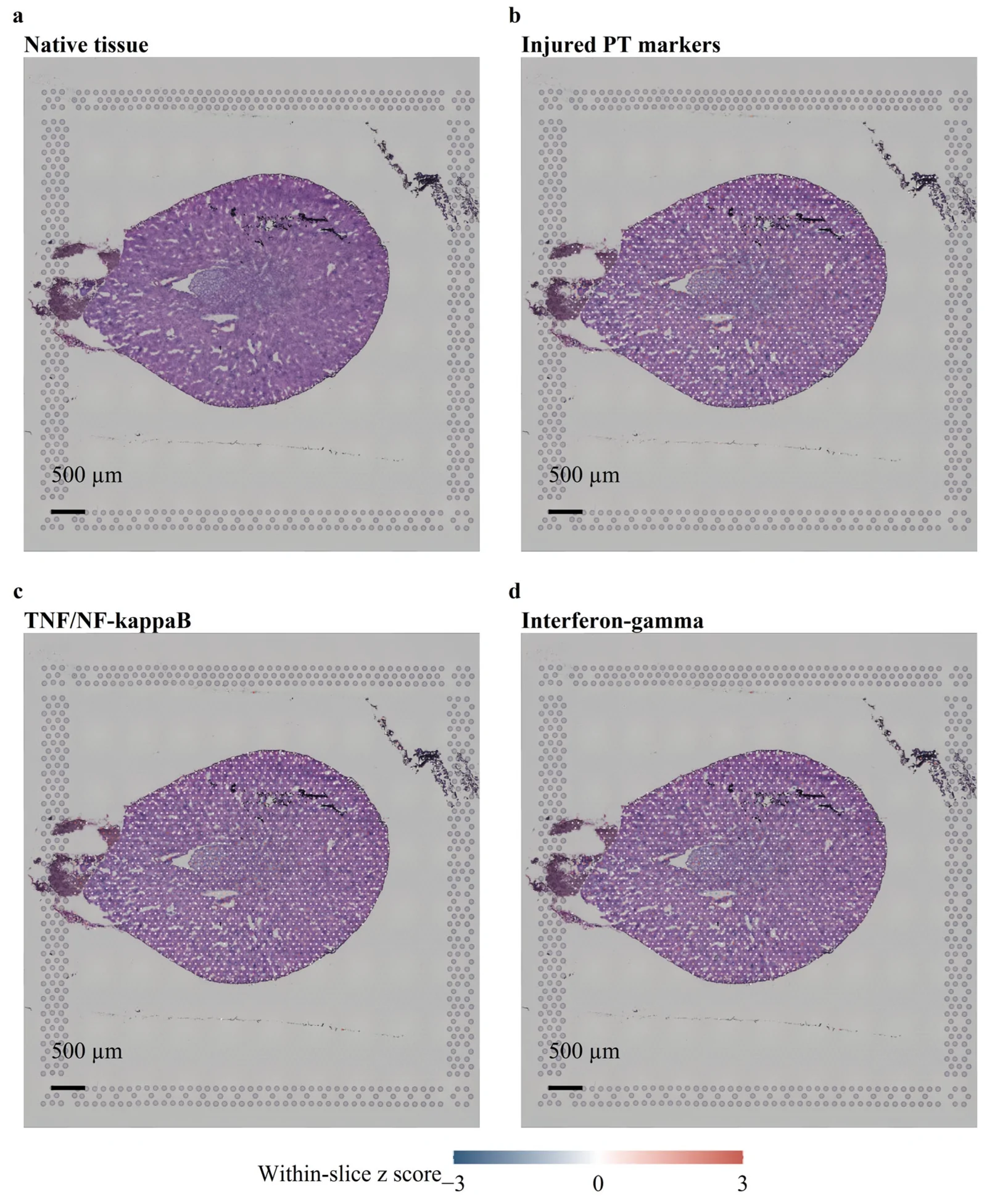
Sham tissue and registered transcriptional modules. (**a**) Native high-resolution tissue image. (**b**–**d**) Injured-PT, TNF/NF-kappaB, and interferon-gamma scores on all 1833 retained spots. Scores are depth-adjusted and standardized within the slice; inflammatory overlays exclude shared injury genes. Full native fields and original tissue colors are preserved. Score opacity is 0.8, saturation is ±3, and scale bars are nominal 500 µm. Source: GSE171406 and Melo Ferreira et al. [22]. Registration and scale derivation are described in Appendix A.8.

**Figure A10:**
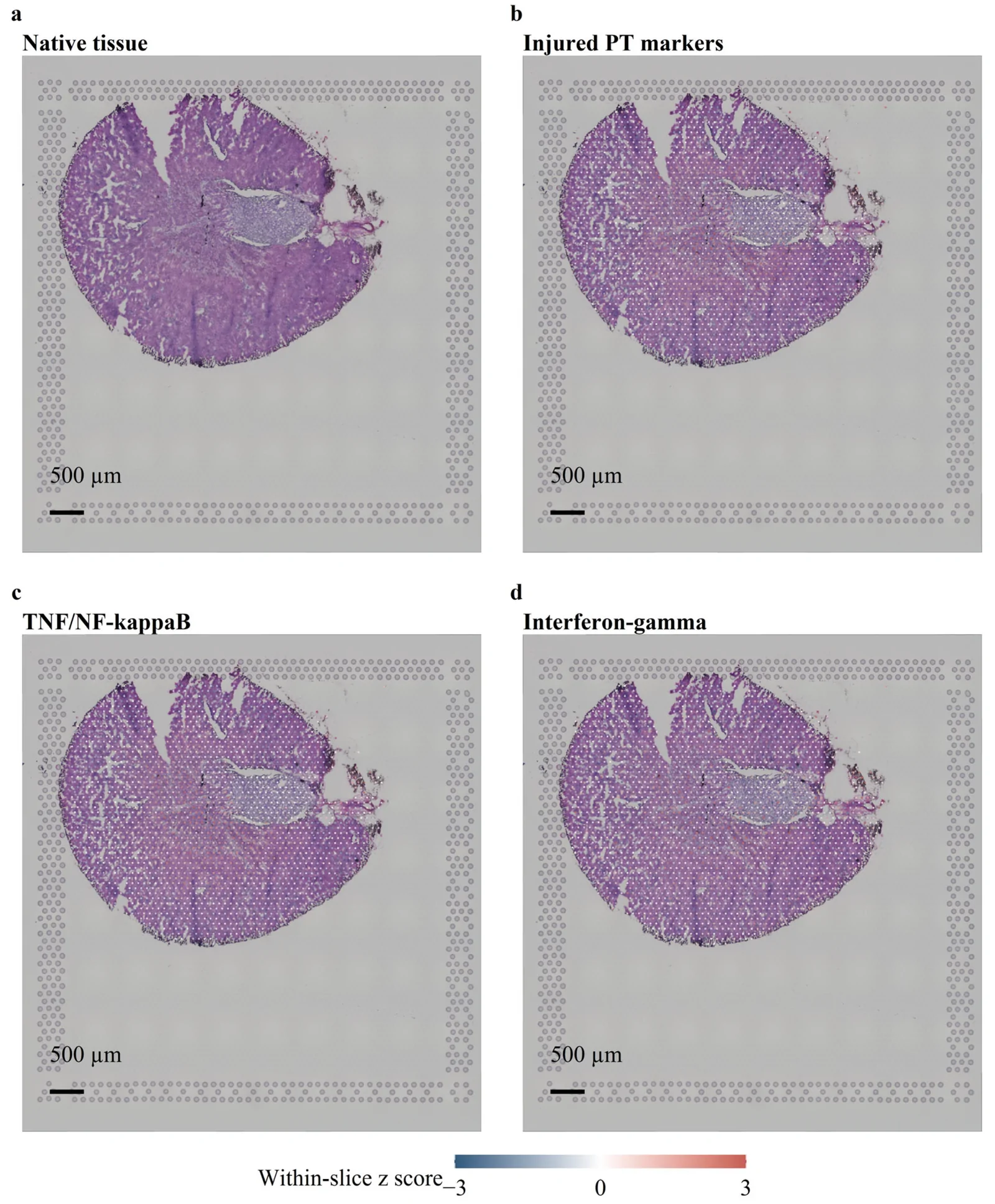
IRI tissue and registered transcriptional modules. (**a**) Native image. (**b**–**d**) Injured-PT, TNF/NF-kappaB, and interferon-gamma scores on all 2063 retained spots. The full native field corresponds to the cropped views in Figure 6. Processing, score scales, and overlay settings follow Figure A9. Scale bars: nominal 500 µm. Source: GSE171406.

**Figure A11:**
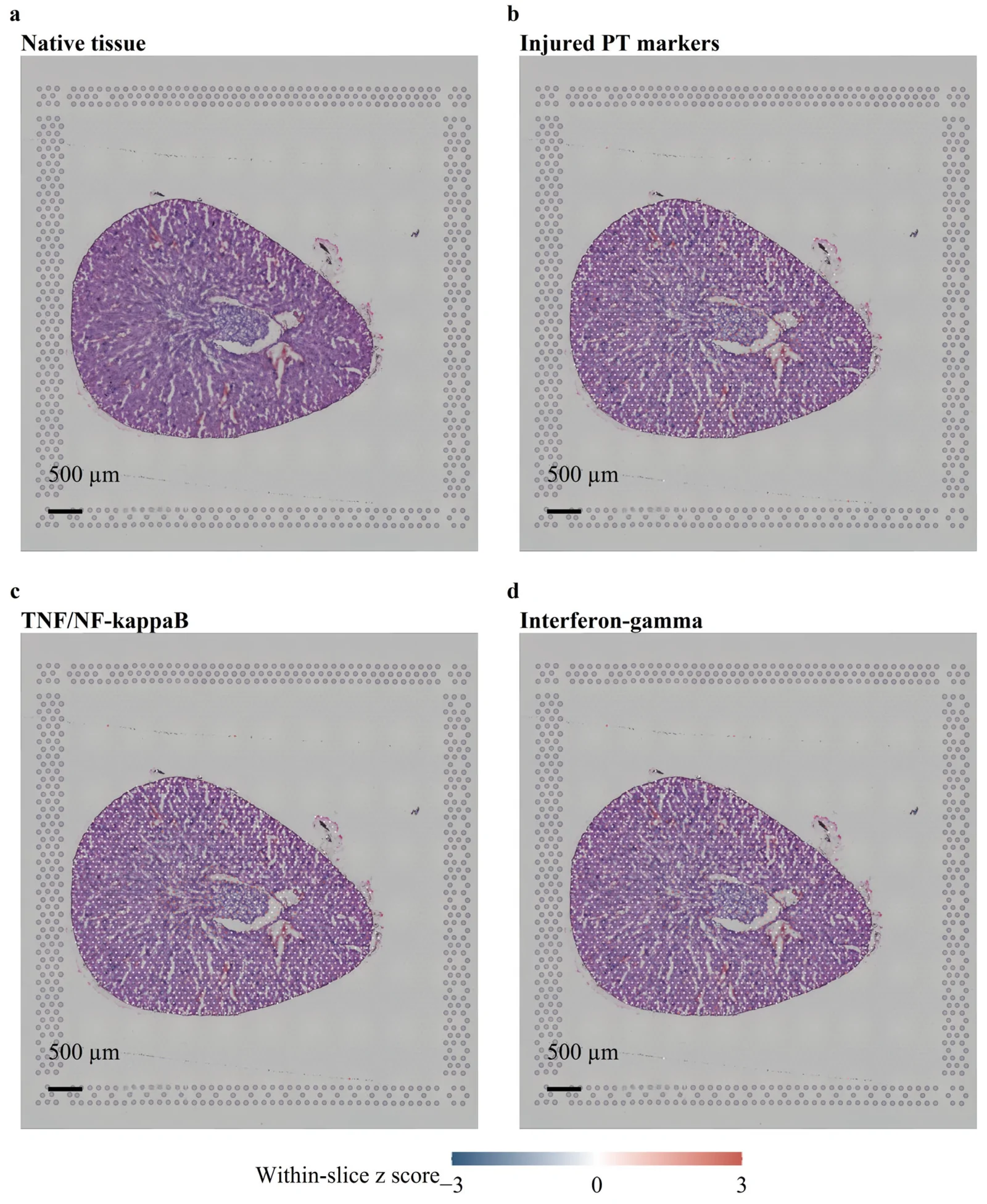
CLP tissue and registered transcriptional modules. (**a**) Native image. (**b**–**d**) Injured-PT, TNF/NF-kappaB, and interferon-gamma scores on all 1888 retained spots. Processing and display settings follow Figure A9. Scale bars: nominal 500 µm. Each slice is standardized independently; equal colors across conditions do not indicate equal absolute expression. Source: GSE171406.

## Data Availability

The public datasets are available from GEO under GSE210622, GSE329941, GSE209610, and GSE171406. Machine-readable Supplementary Tables S1–S5 accompany this manuscript and provide the formal gene-level coordinate, targeted redox-panel results, seven-pathway gene contributions, leading-edge memberships, and pathway statistics. Processed differential statistics, complete pathway results, frozen gene sets, ortholog mappings, sensitivity outputs, and analysis code are available from the corresponding authors on reasonable request. Source tissue images and associated registration files are available from the original spatial dataset. Additional information relevant to this reanalysis may be requested from the corresponding authors.

## Supplementary Materials

The following supporting information can be downloaded at: https://www.mdpi.com/article/10.3390/antiox15101239/s1, Table S1: Formal gene-level coordinate; Table S2: Targeted redox-panel results; Table S3: Seven-pathway gene contributions; Table S4: Leading-edge memberships; Table S5: Complete pathway statistics. Additional supporting methods, analyses, and figures are provided in Appendix A and Appendix B.

## Abbreviations

AKI	acute kidney injury
BH	Benjamini–Hochberg
CKD	chronic kidney disease
CLP	cecal ligation and puncture
EGT	ergothioneine
FDR	false-discovery rate
GEO	Gene Expression Omnibus
GFP	green fluorescent protein
IFN	interferon
IRI	ischemia–reperfusion injury
LISA	local indicators of spatial association
NES	normalized enrichment score
NF-kappaB	nuclear factor kappa B
PT	proximal tubule
TNF	tumor necrosis factor

## Appendix A. Extended Cellular and Pathway Results

### Appendix A.1. Cellular Landscapes and Analysis Populations

The existing cell landscapes provide an overview of the annotated observations underlying the human and EGT studies (Figure A1). The human display contains all 115,936 retained observations from the full 14-library reference, including libraries outside the focal PT contrast. The EGT display retains the previously exported stratified sample of 150,000 from 197,767 cells. No new embedding or further sampling was performed. The inherited “failed-repair PT” label is retained as source annotation; pooled display composition describes these exports and is not a donor-level abundance comparison.

### Appendix A.2. Complete Human Hallmark Landscape

Figure A2 and Figure A3 display all 50 Hallmark pathways. One clustering order was calculated from the two-column NES profiles and then split across the consecutive figures. The ordering brings together pathways with similar enrichment patterns; it does not cluster biological samples.

**Human Hallmark pathways: 1**

**Human Hallmark pathways: 2**

### Appendix A.3. Cross-Species Evidence and Annotation-Marker Sensitivity

The cross-species comparison used the same retained one-to-one ortholog universe for human AKI, mouse disease, and mouse EGT rankings. Under the defined analysis criteria, 31 pathways showed three-way support before cytosolic ribosomal-protein gene removal and seven retained support afterward. These counts are intersections of concordant enrichment calls from related profiles, not independent replication probabilities.

The human focal PT annotation used fixed markers (VCAM1, CCL2, CXCL1, KRT20, CDKN1A, and TGFB2). Removing these six genes from the human ranking and memberships retained concordant support for interferon-alpha, interferon-gamma, IL6–JAK– STAT3, reactive oxygen species, inflammatory-response, KEAP1–NFE2L2, and Reactome interferon-gamma programs. This sensitivity test addresses their direct contribution to enrichment.

**Table A1:** Sample-level cell counts in focal pseudobulks. Counts are retained cells or nuclei entering the sample-level analysis; they are not independent statistical replicates.

Dataset	Group	Samples	Cells Per Sample (Range)
GSE210622	Injury-associated PT (inherited “failed-repair PT” annotation), nephrectomy control	3	40–90
GSE210622	Injury-associated PT (inherited “failed-repair PT” annotation), AKI	7	45–196
GSE329941	Proximal tubule, control	3	23,010–26,325
GSE329941	Proximal tubule, AKI–CKD	3	18,053–22,479
GSE329941	Proximal tubule, AKI–CKD+EGT	3	16,377–20,763

The EGT disease-versus-control and EGT-versus-disease contrasts were extracted from one three-condition edgeR fit. Their arithmetic sum supplies the fitted point estimate for EGT versus control used in the gene-level companion display.

### Appendix A.4. EGT Pathway Landscape and Gene-Level Resolution

All 61 initially supported disease-up/EGT-down pathways appear in Figure A4, Figure A5 and Figure A6, including the 25 that retain support after cytosolic ribosomal-protein gene removal. The retained Hallmark pathways are adipogenesis, DNA repair, E2F targets, mTORC1 signaling, MYC targets V1 and V2, oxidative phosphorylation, p53 pathway, and reactive oxygen species response. Reactome associations include mitochondrial import and translation, respiratory-chain processes, DNA replication and repair, RNA processing, glycan metabolism, and trafficking. Mitochondrial ribosomal genes are distinct from the cytosolic group removed here.

Pathway aggregation and per-gene testing address complementary hypotheses. The pathway result summarizes coordinated ranking across member genes, while the EGT-versus-control effect distribution in Figure 2b provides a fitted gene-level companion view for experimental prioritization.

**EGT-supported pathways: 1**

**EGT-supported pathways: 2**

**EGT-supported pathways: 3**

### Appendix A.5. Sensitivity to Gene Membership and Testing Families

The primary enrichment setting used CAMERA with an assumed inter-gene correlation of 0.01. Gene-membership controls, annotation-degree/rank matching, combined-library correction, and pathway-overlap analyses evaluate the stability and interpretation of the reported pathway patterns.

Correction across a combined Hallmark–Reactome family produced the results in Table A2. The raw probabilities were pooled within each contrast, analysis mode, and method, with the same dual-method criterion applied afterward. This sensitivity changes the correction family while holding rankings and enrichment tests fixed.

The final degree-and-rank calipers identified common-support subsets of 79/80 human and 25/56 EGT ribosomal targets. Maximum-cardinality matching fixed these subsets before the final endpoint evaluation. Randomized matching favored closer eligible pairs using a distance-weighted assignment score with Gumbel perturbations. Every accepted assignment met the stated calipers and feature-balance threshold (maximum absolute standardized mean difference, 0.0995).

Table A3 distinguishes deletion of common-support targets from the conditional full-set analysis. In the former, unmatched ribosomal genes remain unchanged; in the latter, unmatched targets are deleted in every actual and reference run. Human draws comprised 1000 distinct replacement sets, whereas the EGT assignments mapped to four recurring replacement sets. These results define the reference space used for interpreting annotation connectivity.

**Table A2:** Separate-library and combined-library BH sensitivity at CAMERA correlation 0.01. Entries give supported enriched pathways for human PT and supported opposite-direction pathways for EGT.

BH Family	Human Full	Human Removal	EGT Full	EGT Removal
Separate Hallmark/Reactome	187	159	61	25
Combined Hallmark–Reactome	192	165	60	24

**Table A3:** Degree-and-rank-matched removal sensitivity. All counts are retained baseline CAMERA-supported labels at correlation 0.01; baseline denominators are 203 human and 66 EGT pathways. Reference intervals are empirical percentiles from 1000 draws, not biological confidence intervals.

Removal Scope	Profile	Ribosomal	Reference Median	2.5–97.5%
Common support (79/25 genes)	Human	176	161	155–169
Common support (79/25 genes)	EGT	62	57	57–58
Conditional full set (80/56 genes)	Human	168	156	150–162
Conditional full set (80/56 genes)	EGT	26	47	47–47

In the conditional EGT analysis, reference draws retained 47 labels and the observed full-set removal retained 26. Because the conditional background removes unmatched targets from every run, this analysis is interpreted together with the common-support endpoint and the primary number- and rank-matched controls. The paired scopes show how annotation connectivity shapes pathway retention.

Measured-gene complete-linkage cluster counts for thresholds 0.15, 0.20, 0.25, 0.30, and 0.35 were, respectively, 63, 72, 81, 88, and 95 for human full-ranking enrichment; 61, 67, 73, 80, and 86 for human enrichment after removal; and 29, 31, 32, 36, and 38 for the 61 EGT pathways. Leading-edge overlap gave the same qualitative pattern of recurrent gene membership. These descriptive clusters summarize annotation redundancy rather than counts of independent biochemical mechanisms.

### Appendix A.6. Complete Leading-Edge Membership Accounting

Figure A7 preserves all nonempty intersections among the original disease-side and treatment-side leading-edge unions. These broader sets differ from the seven-pathway common-ortholog gene network in the main text.

### Appendix A.7. Complete PT-State Landscape

Figure A8 displays all 160 combinations in the exploratory 20-pathway-by-eight-state panel. Its pathway selection preceded this focused reanalysis, while the primary human and EGT results use complete libraries. The figure preserves the published state framework and highlights heterogeneous within-state responses.

**Four-week PT states: GFP-positive vs. control**

### Appendix A.8. Spatial Registration and Local Testing

Native high-resolution tissue images, barcode positions, and scale factors were obtained from GSE171406 [22]. All 5784 retained barcodes matched source positions, in-tissue flags, and count-file array coordinates. Plots use high-resolution-scaled pixel columns and negative rows. Tissue colors are unchanged, with no contrast enhancement or stitching. Main-text IRI views use a common crop containing all retained spots; Appendix B preserves the full fields. Score overlays use opacity 0.8 and saturation at ±3 within-slice standardized units. Markers are display overlays, not physical capture-spot areas. Nominal 500 µm scale bars use the high-resolution spot diameter multiplied by 500/55, based on the platform specification.

VCAM1 was shared by the injury-marker and interferon-gamma modules and was removed from both in the pairwise disjoint analysis. For map overlays, inflammatory modules exclude shared injury genes; the injury module itself remains intact. Spatial correlations use the separately calculated pairwise disjoint scores. Block-omission analyses retained the original normalization and depth-adjustment models and assess influence on the estimated association.

The spatial graph used six nearest neighbors, distance trimming at 1.5 times the median sixth-neighbor distance, symmetrization, and row standardization. Local indicators of spatial association (LISA) used spdep::localmoran_perm, 999 conditional permutations, no repeat within a row, and deterministic seeds [49]. Two-sided probabilities were twice the folded probabilities, bounded at one. The three modules and three slices formed one BH family of 17,298 testable entries from 17,352 entries; the registered maps provide a complete view of local score organization.

## Appendix B. Native Tissue Images and Complete Overlays

Figure A9, Figure A10 and Figure A11 show the complete native fields and registered overlays for sham, IRI, and CLP. Each condition contributes one slice; colors describe relative transcription within that slice.
